# Baseline pain, fatigue, and sleep quality predict 12-week pain improvement in inflammatory arthritis: retrospective real-world analysis of a digital health application cohort

**DOI:** 10.1007/s00296-026-06105-4

**Published:** 2026-04-10

**Authors:** Dmytro Fedkov, Danylo Yevstifeiev, Oleg Iaremenko, Daria Koliadenko, Liubov Petelytska, Christine Peine, Felix Lang, Abdullah Khalil, Türker Kurt, Stefan Vordenbäumen

**Affiliations:** 1 Department of Internal Medicine #3, Bogomolets National Medical University, 13 Blvd Shevchenka, Kiev, 01032 Ukraine; 2 Medical Center Medical Clinic Blagomed LLC, Kiev, Ukraine; 3 Midaia GmbH, Heidelberg, Germany; 4 Department Rheumatology, St. Elisabeth-Hospital Meerbusch-Lank, Hauptstr. 74-76, 40668 Meerbusch, Germany; 5 Department of Rheumatology, University Hospital Düsseldorf, Medical Faculty of Heinrich Heine University, Düsseldorf, Germany; 6 Hiller Research Center, University Hospital Düsseldorf, Medical Faculty of Heinrich Heine University, Düsseldorf, Germany

**Keywords:** Arthritis, Rheumatoid, Spondylarthropathies, Arthritis, Psoriatic, Digital health, Prediction algorithms, Pain

## Abstract

Despite advances in pharmacotherapy, many patients with inflammatory arthritis (IA) experience residual pain even in remission. Digital health applications (DHAs), when combined with machine learning, may help identify predictors of pain relief in real-world care. To identify predictors of clinically meaningful pain relief in IA using DHA data. We retrospectively analyzed 914 adults with rheumatoid arthritis, psoriatic arthritis, or spondyloarthritis using the Mida Rheuma App. Patients were categorized according to sufficient pain relief, defined as ≥ 30% reduction in pain intensity (visual analogue scale, 0–100) at 12 weeks. Candidate predictors for pain relief included baseline demographics, diagnosis, disease duration, and psychosocial and lifestyle factors: fatigue (Brief Fatigue Inventory, BFI), psychological distress (Patient Health Questionnaire, PHQ-4), sleep and diet quality, physical activity, social support, and app engagement. Multivariable logistic regression and Random Forest models were fitted; non-linear effects were explored using Shapley Additive Explanations. 25.4% of patients achieved ≥ 30% pain reduction. Higher baseline pain intensity was the strongest predictor of response (adjusted odds ratio [OR] 1.59). Fatigue reduced the likelihood of improvement (BFI OR = 0.71), with a SHAP-defined threshold of BFI > 6. Better sleep quality was associated with an increased likelihood of response (OR = 1.23). In exploratory SHAP analyses, the decline in response probability was steepest above BFI of approximately 6. Model discrimination was modest (receiver operating characteristic area under the curve ≈0.61). In IA, pain relief in digital care is associated with baseline pain severity, fatigue, and sleep quality. These findings should be interpreted as hypothesis-generating and require external validation before clinical application.

**Supplementary Information:**

The online version contains supplementary material available at 10.1007/s00296-026-06105-4.

## Introduction

Inflammatory arthritides (IA), including rheumatoid arthritis (RA), psoriatic arthritis (PsA), and spondyloarthritis (SpA), impose a substantial burden on patients' quality of life, primarily due to pain and fatigue [1–5]. While the advent of biologic and targeted synthetic disease-modifying antirheumatic drugs has revolutionized the control of inflammation [4], a significant "efficacy-effectiveness gap" remains in real-world settings [6]. A substantial proportion of patients, up to 20–40% in RA and even higher in PsA, continue to report clinically significant pain despite achieving remission or low disease activity according to inflammatory markers [7–10]. This "residual pain" or "refractory pain" suggests that mechanisms beyond peripheral inflammation, such as central sensitization, sleep disturbance, and maladaptive behavioural patterns, play a critical role in the patient's symptomatic trajectory [11–13].

Addressing this heterogeneity requires a shift from generic treatment protocols toward precision medicine strategies that account for the multidimensional nature of pain [14, 15]. However, traditional clinical predictors (e.g., swollen joint counts, CRP) have shown limited utility in predicting individual pain responses, partly because they fail to capture the lifestyle and psychosocial context in which patients live [16]. Factors such as sleep quality, dietary habits, and psychological distress are increasingly recognized as modifiable drivers of disease impact [17, 18], yet they are rarely collected systematically in routine clinical care or traditional registries.

The digitalization of healthcare offers a novel solution to this data gap. Digital health applications facilitate the collection of high-frequency Real-World Data (RWD), capturing granular fluctuations in Patient-Reported Outcomes (PROs) and behavioural signals that are invisible to episodic clinical visits [19, 20]. Regulatory bodies, including EULAR and the FDA, increasingly recognize the value of such RWD for bridging the gap between controlled trials and clinical practice [21, 22]. Nevertheless, the analysis of these complex, multidimensional datasets presents a methodological challenge. Conventional linear regression models often fail to capture non-linear interactions between variables (e.g., threshold effects of fatigue or "headroom effects" of baseline severity) [23]. In this context, machine-learning algorithms such as Random Forest (RF) can complement conventional regression by capturing non-linear effects and interactions that may be missed by linear models [24–26], while still allowing for interpretation via methods like SHapley Additive exPlanations (SHAP) [27].

Therefore, this study aimed to identify multimodal predictors of clinically meaningful short-term pain relief in a large, real-world cohort of patients with IA using a prescribed digital health application. Unlike previous studies that focused solely on biologic responses, we specifically targeted a patient-centered outcome: a ≥ 30% reduction in pain intensity, which represents a moderate, clinically important improvement according to the Initiative on Methods, Measurement, and Pain Assessment in Clinical Trials (IMMPACT) guidelines [28]. By integrating clinical, demographic, psychosocial, and lifestyle data into a machine learning framework, we sought to determine whether behavioural factors (such as sleep and diet) provide independent predictive value beyond diagnosis and baseline symptom severity.

## Methods

### Study design and patients

This retrospective observational study analyzed anonymized real-world data from 2924 patients with a physician-confirmed diagnosis of inflammatory arthritis. Data were collected between January 2022 and June 2025 from users of the Mida Rheuma App (Midaia GmbH, Germany), a CE-certified medical device (class I) [29]. The application is designed to support patient self-management by providing personalized therapeutic and educational modules. These modules cover nutrition and weight management, physical activity and psychological coaching, sleep and stress hygiene, fatigue management, and medication literacy. All enrolled users have access to structured educational action plans tailored to their profiles.

This manuscript uses the same overarching digital platform as our prior adherence-focused publication [30], but addresses a different endpoint (clinically meaningful pain improvement rather than adherence), a different analytic cohort definition, and distinct clinical questions and interpretations.

Inclusion criteria were: adult patients (≥ 18 years) with a diagnosis of RA, SpA, or PsA, confirmed by a rheumatologist; consent to use data for research purposes; and availability of baseline assessment data. For prediction modelling of 12-week outcomes, the analytical cohort was further refined to include only patients with available 12-week follow-up data on pain intensity (N = 914).

The study was conducted in accordance with the ethical principles outlined in the Declaration of Helsinki (October 2024 revision) and the Good Clinical Practice guidelines. It was approved by the Local Ethics Committee of XXXXX (protocol #XXX, dated XXX). All patients provided digital informed consent before using the Mida Rheuma App. This study is reported in accordance with the STROBE guideline for observational studies and with TRIPOD/TRIPOD-AI aligned recommendations for prediction model reporting (checklists provided in the Appendices S1–S3).

### Assessments and outcome definition

We analyzed a comprehensive set of demographic, clinical, and patient-reported baseline parameters. Demographic variables included age, gender, and time since diagnosis. Clinical characteristics included body mass index (BMI, calculated from self-reported height and weight) and diagnosis. Smoking exposure was recorded as self-reported smoking packs. To reduce sparse cells and maximise statistical power in multivariable modelling, composite diagnoses (e.g., RA with secondary SpA features) were collapsed to the primary clinical phenotype (e.g., RA). For regression analyses, we restricted the sample to participants who self-identified as male or female because the small number of respondents in other gender categories resulted in unstable estimates; however, all gender categories are reported descriptively in the baseline characteristics.

Pain intensity was assessed using a 0–100 mm visual analogue scale (Pain VAS, PPAIN; higher scores indicate more intense pain). Global disease impact was measured with a 0–100 mm patient global assessment (PGADA; higher scores indicate worse overall disease impact). Fatigue was assessed using the Brief Fatigue Inventory (BFI; 0–10), which classifies average fatigue over the previous 24 h as mild (0–3), moderate (4–6) or severe (7–10) [31]. Psychological distress was measured with the four-item Patient Health Questionnaire (PHQ-4; total score 0–12), with conventional severity strata of 0–2 (normal), 3–5 (mild), 6–8 (moderate) and 9–12 (severe); in this study, a cut-off ≥ 6 was used to indicate at least moderate distress in descriptive analyses [32]. In a predefined subsample with additional data, we calculated RAPID3 and BASDAI to benchmark the app-specific indices against established composite measures. RAPID3 (0–30) was computed as the sum of three 0–10 components (physical function, pain, patient global), and disease activity was categorised as near remission (≤ 3), low (> 3–6), moderate (> 6–12) or high (> 12) [33, 34]. BASDAI was scored on a 0–10 scale, as originally developed and validated, with higher scores indicating greater disease activity [35]. In line with current practice, BASDAI ≥ 4 was taken to reflect active disease, consistent with consideration of biologic therapy [36].

To capture the multidimensional nature of patient health, several composite indices were engineered from raw app data. The Physical Activity Score (range 0–20) was calculated as the sum of self-reported frequency scores (0–4) across five exercise domains: strenuous, moderate, strength, flexibility, and sensorimotor exercises. The Social Support Score (range 0–20) was the sum of scores from two items assessing support from 'Family' and 'Friends', each rated on a 0–10 scale. The Diet Quality Score (range 0–100) was a pre-calculated index provided by the app that reflected adherence to anti-inflammatory dietary principles, with higher scores indicating better diet quality [37]. The Sleep Quality Score (range − 4 to + 4, higher values indicate better perceived sleep quality) was derived by subtracting the count of reported sleep problems (each coded as 1 if present: "Can't Sleep", "Wakes Up at Night", "Wakes Up Too Early", "Nightmares") from a general sleep satisfaction score (rated 0–4).

Both the Diet Quality Score and Sleep Quality Score are app-specific composite indices derived from user-entered items and are intended as pragmatic proxies for perceived diet and sleep quality [Appendix S4]. External validation against established reference instruments was not available in this dataset; therefore, these indices should not be interpreted as diagnostic substitutes, and their associations are interpreted cautiously.

App engagement was quantified by two metrics: App Compliance (average adherence to daily tasks) and App Modules Completed (total number of educational/therapeutic modules finished). Both engagement indicators were computed over the full 12-week follow-up period (from baseline to week 12). App Compliance reflects the proportion of assigned daily tasks completed during follow-up, and App Modules Completed reflects the total number of modules completed during the same period.

The primary outcome was defined as a clinically meaningful reduction in pain intensity at 12 weeks. "Response" was operationalized as a ≥ 30% reduction in PPAIN from baseline. This threshold corresponds to a "moderate clinically important improvement" according to the Initiative on Methods, Measurement, and Pain Assessment in Clinical Trials guidelines [28]. We prioritized single-item PROs (PPAIN, PGADA) over composite indices (RAPID3, BASDAI) to maximize sample size, as composite scores exhibited high missingness in this real-world dataset. In a sub-cohort with complete data, PPAIN and PGADA showed strong correlations with RAPID3 and BASDAI (r > 0.70), supporting their pragmatic use as single-item proxies for composite indices in this real-world dataset (Supplementary Figure S1).

### Statistical analysis

We aimed to identify multimodal predictors of 12-week pain response. Engagement indicators were derived from app use during follow-up and are therefore interpreted cautiously as concurrent behavioural markers rather than strict baseline predictors. Given the exploratory nature of the analysis and the potential for complex, non-linear interactions between behavioral and clinical factors, we employed a dual modelling approach using both linear and non-linear algorithms.

Data preprocessing included imputation of missing values using median imputation for continuous variables and mode imputation for categorical variables. Given the exploratory focus and the use of tree-based models, we chose simple single imputation rather than multiple imputation, acknowledging that this may underestimate uncertainty but preserves the full sample for machine-learning analyses. Variables with more than 30% missingness were generally excluded, except for key engineered indices for which missingness was informative or handled by the scoring algorithm. Predictor-level missingness prior to imputation is reported in Supplementary Table S3. Because single imputation can influence non-linear model explanations, SHAP dependence patterns are presented as exploratory and interpreted cautiously. Continuous predictors were standardized (z-scaled) for logistic regression to facilitate comparison of effect sizes (Odds Ratios). For linear regression, predictors were kept in their original units to ensure clinical interpretability of the coefficients (β). For continuous predictors, ORs are reported per 1-SD increase to facilitate comparison of effect sizes across scales.

First, for the machine learning analysis, we trained a Random Forest classifier (scikit-learn, v1.2.2) using bootstrap aggregating with 100 decision trees. To address the class imbalance (25.4% responders), class weights were adjusted inversely proportional to class frequencies. We utilized default hyperparameters to prevent overfitting given the sample size: the model used the Gini impurity criterion, no maximum depth constraint (nodes expanded until all leaves were pure or contained less than 2 samples), and a minimum of 1 sample per leaf. To ensure reproducibility, the random number generator was initialized with a fixed seed. Additionally, a Random Forest Regressor with equivalent parameters (using Mean Squared Error criterion) was trained to predict the continuous magnitude of pain reduction. Thus, RF models served as our primary tools for prediction and exploration of non-linear effects, whereas logistic and linear regression models were used as complementary, more interpretable parametric models to quantify effect sizes.

Model performance was evaluated using Repeated Stratified 5 × 2 Cross-Validation, a robust method for estimating generalization error and confidence intervals. We reported the Area Under the Receiver Operating Characteristic Curve (ROC AUC) and sensitivity. Calibration was assessed using a calibration curve (Supplementary Figure S2). Clinical net benefit across decision thresholds was examined using decision curve analysis (Supplementary Figure S3). Feature importance was assessed using SHAP values, which quantify each feature's contribution to the prediction. SHAP summary plots and dependence plots were generated to visualize the directionality and non-linear patterns of effects for continuous variables such as fatigue, disease duration, and BMI.

Second, to quantify linear associations and provide clinically interpretable effect sizes, we fitted a multivariable Logistic Regression model for the binary response outcome. Results are reported as Adjusted Odds Ratios (ORs) with 95% Confidence Intervals (CIs). A multivariable Linear Regression was also performed to estimate the associations with the magnitude of pain reduction (ΔPPAIN%), reporting beta coefficients (β).

All models were adjusted for age, sex, diagnosis, disease duration, and baseline clinical/psychosocial scores. In the primary multivariable models, PGADA was excluded to avoid multicollinearity, given its very strong correlation with PPAIN (r > 0.7).

In a prespecified sensitivity analysis, baseline pain was excluded from the predictor set to assess whether behavioral and psychosocial factors retain predictive value independent of baseline severity. In an additional sensitivity analysis, models were repeated in the subgroup with clinically relevant baseline pain (PPAIN ≥ 30 mm) to reduce floor effects in a percentage-change endpoint. In a second set of sensitivity analyses, all multivariable logistic and linear regression models were repeated (i) in the subset of patients with complete data for disease-activity classification, additionally adjusted for harmonised disease-activity categories, and (ii) in the subgroup of patients classified as being in remission or low disease activity at baseline, to assess robustness to heterogeneity in inflammatory burden. These disease-activity–adjusted sensitivity models were based on the subset with complete data for disease-activity classification (N = 562) and are reported in Supplementary Table S1.

All statistical analyses were performed using Python (v3.10) with the scikit-learn and statsmodels libraries. A two-sided *p*-value < 0.05 was considered statistically significant.

## Results

The source cohort comprised 2,924 registered app users. The predictive cohort comprised 914 users with week-12 pain follow-up. Baseline characteristics of included versus excluded eligible users (those without week-12 pain) are summarized in Supplementary Table S4, indicating potential selection related to follow-up availability. Compared to excluded users, the included cohort was older (46.86 ± 12.78 vs. 44.28 ± 13.31 years, *p* < 0.001) and predominantly female (80.1% vs. 76.1%, *p* < 0.001). Included participants exhibited a lower baseline disease burden, characterized by reduced pain intensity (51.62 ± 25.11 vs. 54.93 ± 25.76, *p* = 0.002), patient global assessment (56.63 ± 25.01 vs. 59.53 ± 25.48, *p* = 0.005), and fatigue levels (4.98 ± 1.98 vs. 5.32 ± 1.93, *p* < 0.001). Furthermore, while overall app compliance was comparable (*p* = 0.192), active engagement was markedly higher in the retained group, as evidenced by a greater number of completed educational modules (5.65 ± 6.44 vs. 1.77 ± 1.63, *p* < 0.001). The extent of missing data across candidate predictors prior to imputation is summarized in Supplementary Table S3.

Overall, 232 patients (25.4%) were categorized with a clinically meaningful ≥ 30% reduction in pain at the 12-week follow-up. At baseline, responders reported significantly higher pain intensity (55.9 ± 23.6 vs 50.2 ± 25.5, *p* = 0.002) but lower fatigue levels (4.7 ± 1.9 vs 5.1 ± 2.0, *p* = 0.007) and better sleep quality scores (0.8 ± 1.8 vs 0.4 ± 1.7, *p* = 0.003) compared to non-responders. In contrast, demographic profiles (e.g., age *p* = 0.092) and diagnostic distributions (*p* = 0.509) were broadly similar between groups (Table 1).

**Table 1:** Baseline demographic, clinical, and psychosocial characteristics of the study population stratified by 12-week pain response status

Characteristic^1^	Non-Responders (N = 682)	Responders (N = 232)	*p*-value^2^
Demographics			
Age, years	46.5 ± 12.9	48.1 ± 12.4	0.092
Male sex, n (%)	135 (19.8%)	47 (20.3%)	0.954
Disease duration, years	8.7 ± 9.0	8.1 ± 9.4	0.364
Body mass index, kg/m^2^	26.9 ± 6.0	26.7 ± 5.5	0.612
Smoking, packs	1.8 ± 1.1	1.9 ± 1.2	0.292
Diagnosis, n (%)			
Rheumatoid arthritis	389 (57.0%)	140 (60.3%)	0.509
Spondyloarthritis	191 (28.0%)	64 (27.6%)	0.509
Psoriatic arthritis	102 (15.0%)	28 (12.1%)	0.509
Clinical symptoms			
Pain intensity (PPAIN), 0–100	**50.2 ± 25.5**	**55.9 ± 23.6**	**0.002**
Disease activity (PGADA), 0–100	56.0 ± 25.2	58.6 ± 24.3	0.151
Fatigue (BFI), 0–10	**5.1 ± 2.0**	**4.7 ± 1.9**	**0.007**
Psychosocial & lifestyle			
Psychological distress (PHQ-4), 0–12	4.1 ± 2.6	3.8 ± 2.7	0.167
Sleep quality score, − 4 to + 4	**0.4 ± 1.7**	**0.8 ± 1.8**	**0.003**
Diet quality score, 0–100	69.4 ± 15.6	71.5 ± 15.7	0.083
Social support score, 0–20	13.5 ± 4.2	14.0 ± 4.2	0.169
Physical activity score, 0–20	7.1 ± 4.9	6.9 ± 4.8	0.545
App engagement			
App compliance, %	52.5 ± 23.8	50.3 ± 22.9	0.264
Modules completed, n	5.6 ± 6.3	5.8 ± 6.8	0.741

^1^Data are presented as mean ± standard deviation for continuous variables and n (%) for categorical variables

^2^*p*-values were calculated using the Student’s t-test for continuous variables and Pearson’s Chi-squared test for categorical variables

Bold values indicate statistical significance (*p* < 0.05). Response is defined as ≥ 30% reduction in pain intensity at 12 weeks

PPAIN, Patient Pain Assessment Intensity; PGADA, Patient Global Assessment of Disease Activity; BFI, Brief Fatigue Inventory; PHQ-4, Patient Health Questionnaire-4

In the multivariable analysis, we evaluated predictors of both the likelihood of achieving a clinically meaningful response (≥ 30% improvement; logistic regression) and the magnitude of pain reduction (linear regression and Random Forest). The Random Forest Regressor model, predicting the magnitude of pain improvement, demonstrated moderate explanatory power (R2≈0.50), indicating that the model captured a substantial proportion of the variance in pain improvement. The Logistic Regression model predicting the binary response achieved an ROC AUC of 0.614 (calibration and decision-curve analyses are provided in Supplementary Figures S2 and S3). The RF classifier achieved a comparable ROC AUC of 0.613, indicating that non-linear modelling improved the interpretability of complex patterns (via SHAP) without a substantial gain in discriminative performance over the simpler logistic regression model. Model performance was similar across conventional regression and Random Forest approaches, indicating limited incremental discrimination from machine learning in this setting. Explainability analyses (SHAP) are presented as exploratory and hypothesis-generating; apparent nonlinearities (e.g., fatigue thresholds) should not be interpreted as clinically actionable cutoffs without external validation and prospective testing.

**Fig. 1:**
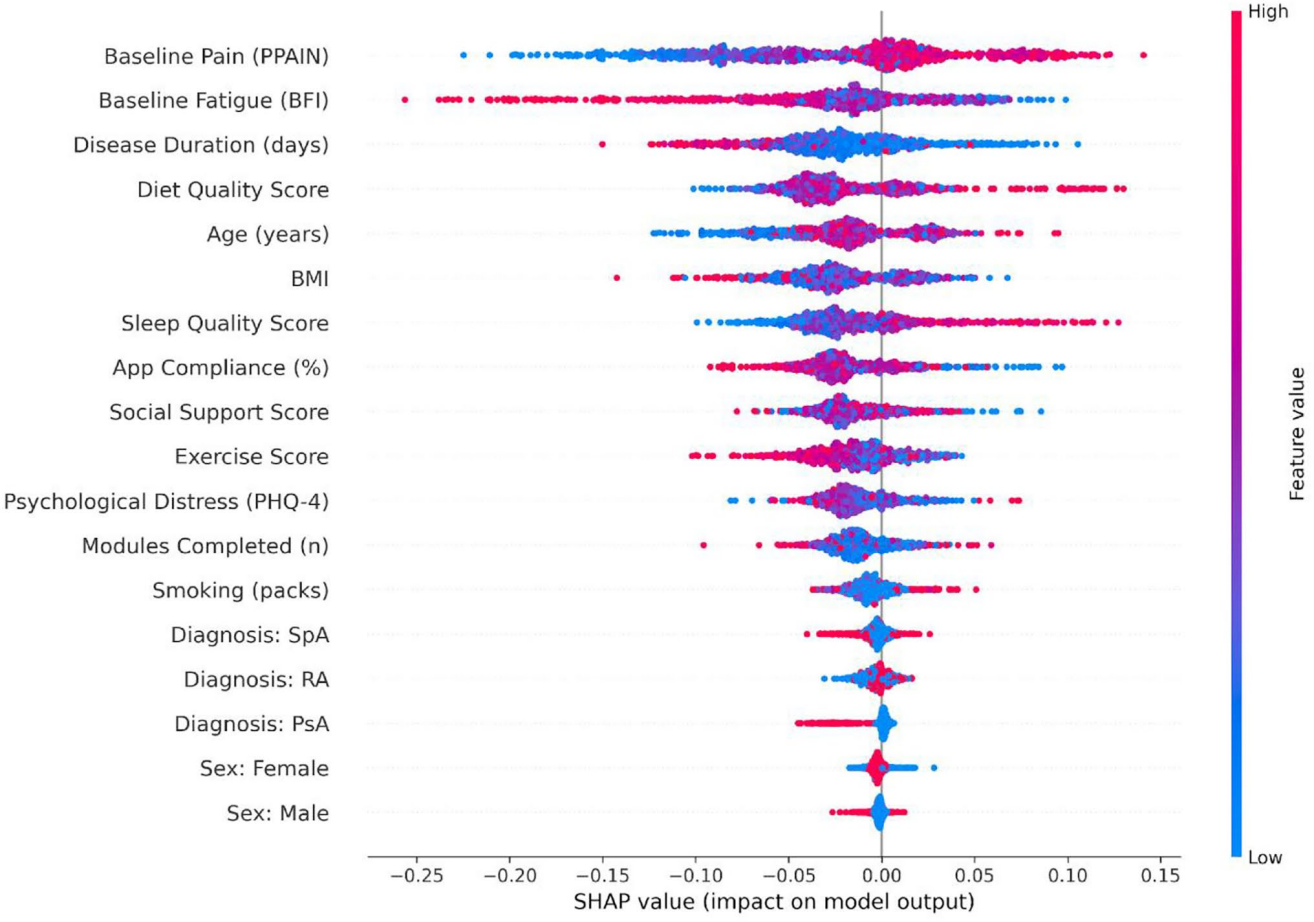
SHAP summary plot illustrating the hierarchy and directionality of predictors for 12-week pain response. The plot displays SHAP values for each feature (model predictor), ranked by their global importance (mean absolute SHAP value). Each point represents an individual patient. The horizontal position indicates the direction and magnitude of that patient's feature contribution to the predicted probability of being categorized with a clinically meaningful ≥ 30% pain reduction. The colour denotes the patient-specific feature value (red = higher values of the predictor, blue = lower values), explaining why points within the same feature vary in colour. Global importance reflects the overall contribution of the feature across all patients, whereas colour reflects the individual feature value for each data point.* PPAIN* patient pain assessment intensity (0–100),* BFI* brief fatigue inventory (0–10),* BMI* body mass index (kg/m2),* PHQ4* patient health questionnaire-4 (0–12),* PsA* psoriatic arthritis,* RA* rheumatoid arthritis,* SpA* spondyloarthritis

Figure 1 displays the contribution of all features to the model's prediction based on mean absolute SHAP values. The strength and directionality of the associations from both logistic and linear regression models are presented in Table 2.

**Table 2:** Adjusted predictors of 12-week clinical response (pain intensity)

Predictor	Binary response (≥ 30% improvement) (logistic regression)		Magnitude of improvement (ΔPPAIN%) (linear regression)	
	Adjusted OR (95% CI)	*p*-value	β coefficient (95% CI)	*p*-value
Baseline clinical				
Baseline PPAIN (0–100)	**1.59** (1.32 to 1.93)	**< 0.001**	**2.98** (2.59 to 3.37)	**< 0.001**
Baseline fatigue (BFI, 0–10)	**0.71** (0.58 to 0.88)	**0.001**	− **8.19** (-13.97 to -2.41)	**0.006**
Psychosocial & lifestyle				
Sleep quality Score (-4 to + 4)	**1.23** (1.04 to 1.46)	**0.018**	**5.71** (0.20 to 11.22)	**0.042**
Diet quality Score (0–100)	1.16 (0.98 to 1.37)	0.100	0.42 (− 0.14 to 0.97)	0.146
Social support Score (0–20)	0.97 (0.82 to 1.15)	0.745	0.55 (− 1.63 to 2.72)	0.622
Demographics				
Age (years)	1.16 (0.98 to 1.38)	0.095	**0.78** (0.05 to 1.50)	**0.036**
Disease duration (years)	0.88 (0.74 to 1.04)	0.144	0.00 (− 0.00 to 0.00)	0.313
Gender (male vs female)	1.04 (0.70 to 1.56)	0.815	− 15.51 (− 37.15 to 6.12)	0.160
Diagnosis (ref: PsA)				
RA	1.31 (0.81 to 2.11)	0.269	7.49 (− 17.44 to 32.42)	0.556
SpA	1.41 (0.83 to 2.38)	0.209	16.23 (− 11.07 to 43.53)	0.244
App usage				
App compliance	0.89 (0.75 to 1.06)	0.195	− **0.95** (− 1.40 to − 0.50)	**< 0.001**
Modules completed	1.07 (0.91 to 1.26)	0.428	− 0.39 (− 2.00 to 1.22)	0.634

Results from two separate multivariable regression models adjusted for all listed covariates simultaneously. Bold values indicate statistical significance (*p* < 0.05)

Binary Response (left): logistic regression predicting the likelihood of achieving a clinically meaningful response (≥ 30% reduction in PPAIN). Values are Adjusted Odds Ratios (OR) with 95% Confidence Intervals (CI). For continuous predictors, ORs are standardized (per 1 SD increase) to facilitate comparison of effect sizes; for categorical predictors, ORs represent the effect relative to the reference group. An OR > 1.0 indicates a higher likelihood of response

Magnitude of Improvement (right): linear regression predicting the percentage change in pain intensity (continuous outcome). Values are unstandardized Beta (β) coefficients. A positive β indicates a greater percentage improvement (larger drop in pain) for every 1-unit increase in the predictor; a negative β indicates less improvement

Reference categories: Psoriatic Arthritis (PsA) for diagnosis; Female for gender

*PPAIN* patient pain assessment intensity,* BFI* brief fatigue inventory,* RA* rheumatoid arthritis,* SpA* spondyloarthritis

**Fig. 2:**
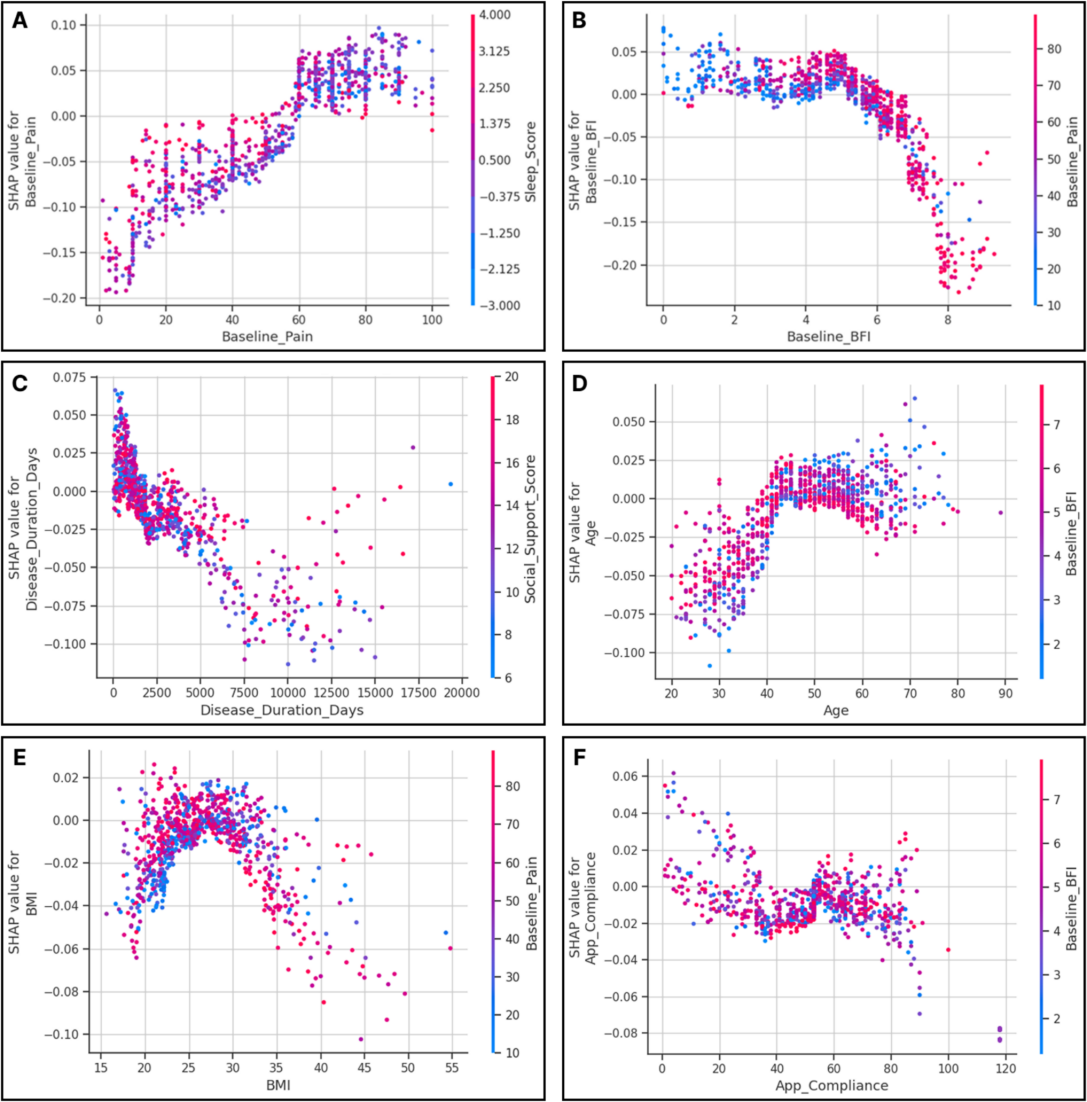
SHAP dependence plots for top continuous predictors. These plots visualize the nonlinear relationships between specific features and their impact on the predicted response likelihood (SHAP value, y-axis). **A** Baseline Pain shows a positive linear trend, consistent with regression to the mean. **B** Fatigue (BFI): exhibits a threshold effect, with BFI scores > 6 associated with a sharp decrease in response probability. **C** Disease Duration: shows a general inverse relationship, with increased variance observed at extreme chronicity (> 20 years). **D** Age: reveals a nonlinear benefit, with the most substantial positive contribution observed in the 40–60-year range. **E** BMI: shows a non-linear effect, peaking in the overweight range (25–30). **F** App Compliance: suggests an optimal benefit window in the moderate range (55–70%). Scatter points are coloured by interacting variables. Abbreviations: BFI, Brief Fatigue Inventory (0–10); BMI, Body Mass Index (kg/m2)

**Fig. 3:**
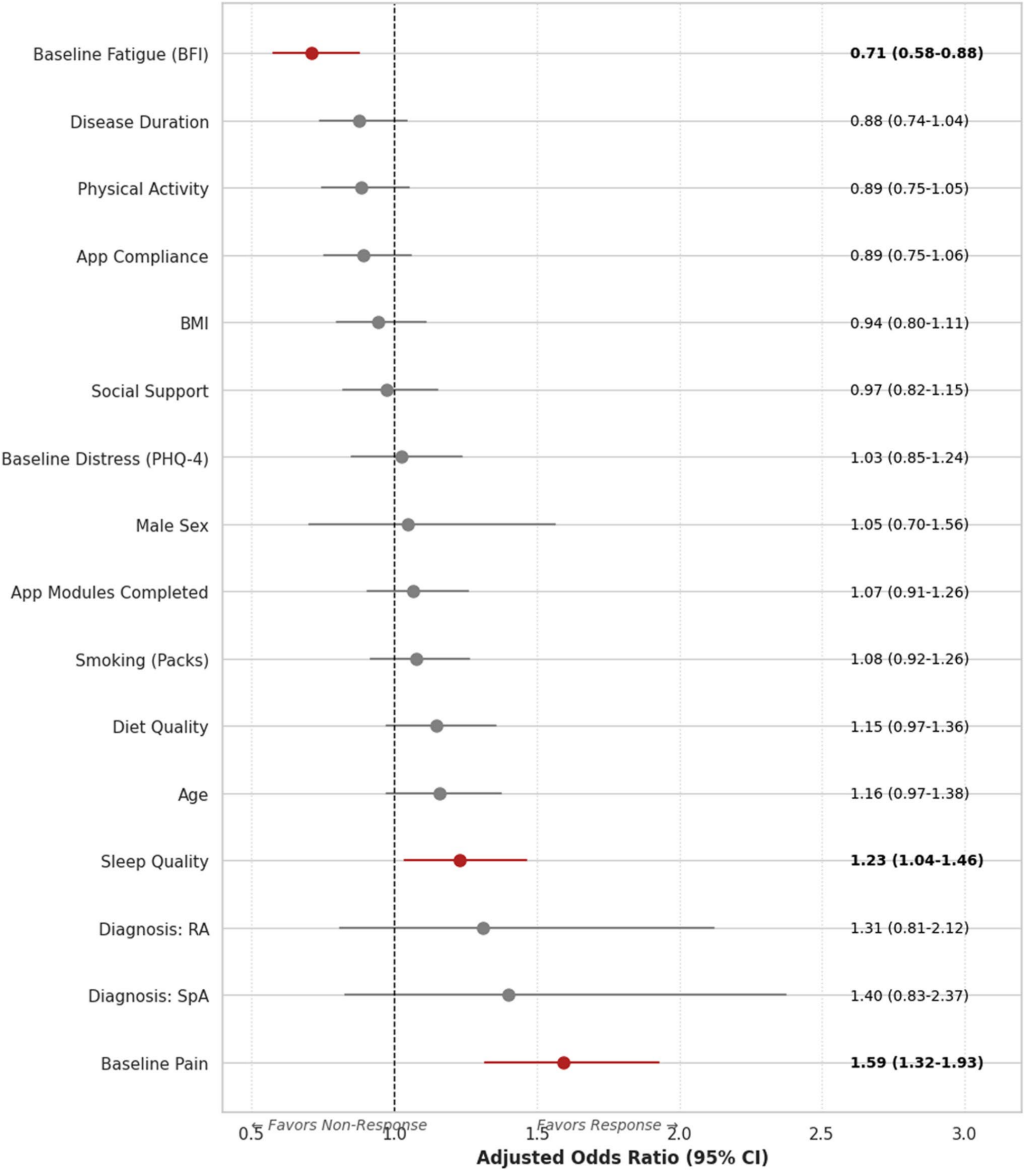
Forest plot of adjusted Odds Ratios for achieving clinically meaningful pain relief. Results from the multivariable Logistic Regression model adjusted for all covariates. Points represent the Adjusted Odds Ratio for achieving a ≥ 30% reduction in pain; horizontal lines represent 95% Confidence Intervals. The vertical dashed line at OR = 1 indicates no effect. Factors to the right (OR > 1) are associated with a higher likelihood of response, while factors to the left (OR < 1) are associated with a lower likelihood.* BFI* brief fatigue inventory (0–10),* BMI* body mass index (kg/m2),* PHQ-4* patient health questionnaire-4 (0–12),* RA* rheumatoid arthritis,* SpA* spondyloarthritis

Higher baseline pain intensity was the strongest consistent predictor across all models. It significantly increased the odds of achieving a response (Adjusted OR 1.59, 95% CI 1.32 to 1.93, *p* < 0.001) and was associated with a larger magnitude of percentage improvement (β = 2.98, 95% CI 2.59 to 3.37, *p* < 0.001) (Fig. 2A, Fig. 3). This reflects the regression to the mean, where patients with higher initial severity have a greater mathematical potential for improvement.

Conversely, Baseline Fatigue (BFI) emerged as a major negative contributor (Adjusted OR 0.71, 95% CI 0.58 to 0.88, *p* = 0.001). In the linear model, each unit increase in fatigue score was associated with an 8.2% decrease in the magnitude of pain relief (β = − 8.19, 95% CI -13.97 to -2.41, *p* = 0.006). Random Forest SHAP analysis revealed a distinct non-linear threshold for fatigue: the negative impact on response became pronounced at BFI scores > 6, with values sharply declining and reaching minimal levels around BFI 8, suggesting that severe fatigue may act as a "blocker" for behavioral interventions (Fig. 2B). Together with disease duration, this delineates a "low-energy / chronic disease" phenotype that may be less responsive to digital self-management, even after accounting for baseline pain.

Sleep Quality was a significant independent predictor of better outcomes (Adjusted OR 1.23, 95% CI 1.04 to 1.46, *p* = 0.018; β = 5.71, 95% CI 0.20 to 11.22, *p* = 0.042), acting as a resilience factor (Fig. 3). Diet quality showed a positive trend (Adjusted OR 1.16, 95% CI 0.98 to 1.37, *p* = 0.100) but did not reach statistical significance in the fully adjusted model.

We analyzed the influence of demographics and app engagement beyond simple linear associations. Older age was associated with a significantly larger magnitude of improvement (β = 0.78, 95% CI 0.05 to 1.50, *p* = 0.036); the SHAP analysis indicated a significant nonlinear effect, with the positive predictive contribution becoming stable and strong for patients aged 40–60 years (Fig. 2D). Similarly, Body Mass Index (BMI) showed a parabolic effect, with the highest probability of response in the overweight range (BMI 25–30). In contrast, values outside this range were associated with a negative impact (Fig. 2E). Disease duration generally showed an inverse relationship, with predictive dispersion increasing significantly beyond 7500 days (approximately 20 years) (Fig. 2C). Regarding diagnosis, patients with SpA demonstrated a trend toward higher odds of response (Adjusted OR 1.41, 95% CI 0.83 to 2.38, *p* = 0.209) compared to PsA (reference) and RA (Adjusted OR 1.31, 95% CI 0.81 to 2.11, *p* = 0.269) (Fig. 3). However, Random Forest SHAP analysis indicated that this "diagnosis effect" was attenuated when accounting for age and activity levels.

We observed a divergence between the quantity of content consumed and the consistency of use. While the total number of completed app modules had only a minor impact, overall App Compliance (regular adherence to daily tasks) showed a substantially larger effect in the SHAP analysis (Fig. 1). This indicates that consistent engagement is the primary driver of clinical benefit. SHAP analysis further revealed strong positive predictive values for compliance in the moderate range (55–70%) (Fig. 2F). Interestingly, in the linear model, higher compliance was associated with a slightly smaller magnitude of improvement (β =  − 0.95, 95% CI − 1.40 to − 0.50, *p* < 0.001), which likely reflects reverse causality, whereby non-responders engage more intensively with the app in an attempt to achieve relief. The influence of App Compliance and other non-linear demographic effects was independent of baseline pain.

To assess whether the identified predictors remained relevant after controlling for the dominant effect of baseline severity, we repeated the modelling while excluding baseline pain intensity. In this reduced Random Forest model, predictive discrimination dropped significantly (ROC AUC 0.547, SD 0.028), indicating that baseline pain is a major driver of overall model performance. Nevertheless, in the adjusted logistic regression within this sensitivity analysis, Sleep Quality Score remained a statistically significant independent predictor (Adjusted OR 1.21, 95% CI 1.02 to 1.43, *p* = 0.032). This confirms that better sleep is associated with a higher likelihood of response, regardless of baseline pain severity, supporting its status as an independent resilience factor. Baseline fatigue showed a similar negative trend (Adjusted OR 0.89) but lost statistical significance after adjustment for pain severity, likely due to the strong correlation between pain and fatigue.

A comprehensive comparison of feature importance between the classification and regression tasks is provided in Supplementary Figure S4. Notably, although baseline pain intensity dominated the regression model (34.2% importance due to the mathematical headroom effect), baseline fatigue (BFI) emerged as the single most important predictor for the binary classification of response (10.7%), surpassing pain intensity. This highlights fatigue as a critical, distinct barrier to achieving a clinically meaningful outcome.

After adjustment for harmonised disease activity categories, the direction and magnitude of associations between key predictors and the probability of being categorized with a clinically meaningful ≥ 30% reduction in pain remained largely unchanged (Supplementary Table S1 and Supplementary Figure S5). Patients classified as in the "Remission/Low activity" category at baseline had significantly higher odds of achieving a pain response than those with High activity (Adjusted OR 2.36, 95% CI 1.03–5.41, *p* = 0.043), independent of baseline pain levels. This confirms that the observed predictors are not merely proxies for disease activity.

In sensitivity analyses restricted to the subgroup of patients in remission or low disease activity at baseline (N = 108), the main predictive patterns were preserved. However, confidence intervals widened as expected due to the reduced sample size (Supplementary Table S2 and Supplementary Figure S6). Crucially, the importance of Sleep Quality became more pronounced in this subgroup, emerging as the strongest significant predictor (Adjusted OR 2.08, 95% CI 1.13–3.85, *p* = 0.019). Additionally, the number of App Modules Completed showed a stronger trend toward significance (OR 1.67, *p* = 0.079) than in the whole cohort, suggesting that behavioral engagement and sleep hygiene may be particularly effective drivers of pain relief when systemic inflammation is controlled.

## Discussion

Compared with our earlier pilot evaluation of the Mida Rheuma App and our recent adherence-focused analysis, the present work addresses a distinct clinical question: the improvement in residual pain, defined by a patient-centred threshold (> = 30% reduction), and its baseline predictors in a larger routine-use cohort. We further add explainable modelling (SHAP) to generate hypotheses about potentially modifiable factors such as fatigue and sleep, while explicitly acknowledging modest discrimination and the need for external validation.

Our extensive real-world study of 914 patients with IA demonstrates that achieving a clinically meaningful reduction in pain (≥ 30%) is driven by a complex interplay of baseline symptom severity, psychosocial resilience, and disease phenotype. Our multivariable machine learning analysis revealed three key findings. First, we confirmed a strong regression-to-the-mean pattern, in which higher baseline pain intensity was the most potent positive predictor of response magnitude. Second, we identified baseline fatigue as a major barrier to improvement, with a distinct non-linear threshold (BFI > 6) beyond which the probability of pain relief drops significantly. Third, sleep quality emerged as an independent resilience factor, improving the odds of response regardless of baseline pain or diagnosis. While patients with SpA showed a trend toward better outcomes compared to RA and PsA, our analysis suggests this may be partly mediated by younger age and specific engagement patterns rather than diagnosis alone.

Consistent with the "Law of initial value" observed in other chronic conditions [38], baseline pain intensity was the strongest predictor of response in our models. This finding reflects the mathematical reality that patients with higher initial scores have greater potential for percentage-based improvement. However, it is crucial to distinguish this from regression to the mean. By filtering our cohort for clinically significant baseline pain (≥ 30 mm) and adjusting for confounders, we isolated a signal suggesting that high inflammatory pain is inherently more "responsive" to intervention than lower-level, potentially nociplastic pain. This aligns with findings from the FINCH trials, where higher baseline severity predicted greater absolute reductions [39]. Conversely, the observation that patients with Psoriatic Arthritis had the lowest likelihood of response (serving as the reference group against which SpA and RA showed trend-level superiority) may reflect a higher burden of central sensitization [40] and enthesitis-driven pain [41], which are known to be less responsive to standard management than synovitis [42].

A critical insight from our Random Forest analysis is the negative predictive role of fatigue. The strong inverse relationship between baseline fatigue and pain relief suggests that fatigue is not merely a symptom but a rate-limiting factor in recovery. Identifying a threshold of BFI > 6 provides a concrete clinical marker: patients exceeding this level may require targeted fatigue-management interventions (e.g., cognitive-behavioural therapy for insomnia or specific aerobic exercise) [43] before they can effectively engage in pain-relief behaviours. This supports the concept of a "low-energy / chronic disease" phenotype that is resistant to digital self-management alone [44]. A potential practical implication is the identification of a high-fatigue phenotype with a lower probability of pain improvement; this observation is hypothesis-generating and supports prospective testing of targeted support pathways rather than immediate clinical decision support. In this context, “prioritisation” refers to the emphasis on fatigue- and sleep-focused module domains within the app’s tailored recommendations for users with high fatigue or poor sleep.

In contrast to fatigue, better sleep quality acted as a significant positive predictor. This finding validates the bidirectional relationship between sleep and pain regulation. Good sleep likely functions as a physiological resource, restoring descending pain inhibitory pathways and enhancing the patient's capacity to cope with symptoms [45, 46]. Similarly, the positive trend for diet quality reinforces the emerging role of anti-inflammatory nutrition in symptom control [47, 48]. Importantly, our sensitivity analysis confirmed that the protective effect of sleep persists even when controlling for (or excluding) baseline pain severity. This suggests that sleep optimization is not merely a byproduct of lower pain but an independent therapeutic target that may enhance the efficacy of other interventions [49].

Patients with SpA had higher odds of response than the reference group. Several mechanisms may explain this signal. First, SpA is particularly responsive to structured physical activity, mobilization, and posture-focused exercise programmes, which are core components of non-pharmacological management and have demonstrated substantial effects on pain and stiffness in this population [50–52]. In contrast, pain in RA and PsA frequently includes a larger structural and neuropathic component, limiting the magnitude of improvement achievable through behavioural interventions alone [53–55]. Second, SpA cohorts tend to be younger and have fewer comorbidities [56, 57], which may facilitate engagement in exercise and behavioural change. Finally, while inflammatory back pain in SpA often shows rapid improvement with movement, mechanical pain in established RA/PsA may sometimes worsen with activity [58]. However, our RF SHAP analysis indicated that this effect was attenuated when accounting for age and activity levels, suggesting that the "SpA advantage" is partly demographic and behavioural. Fully stratified multivariable models within each diagnosis were not performed because small subgroup sample sizes (especially PsA) would yield unstable estimates; diagnosis-specific prediction will require larger cohorts. Thereby, findings should be interpreted as hypothesis-generating, warranting further condition-specific trials.

This study is predictive and hypothesis-generating; engagement indicators were computed over the follow-up period and may reflect symptom change (reverse causality), so they should be interpreted as co-occurring behavioural signals rather than as baseline predictors or causal drivers of improvement. Our analysis also revealed a divergence between the quantity of content consumed and the consistency of use. While the total number of completed app modules had minimal impact, overall app compliance (regularity of use) was a stronger driver of outcomes. This suggests that the dose of a digital intervention is defined not by information volume but by the consistency of the behavioural nudge. Interestingly, the negative linear association between compliance and the magnitude of improvement likely reflects reverse causality (non-responders engaging more intensively to find relief), a phenomenon often observed in real-world practice.

To reduce confounding by inflammatory burden, we harmonised disease activity across RA, SpA and PsA and adjusted all prediction models accordingly. Sensitivity analyses restricted to patients in remission or low disease activity yielded similar patterns, indicating that the findings are not solely driven by baseline inflammation. In these disease-activity–adjusted models, the direction and magnitude of the main predictors remained essentially unchanged, whereas sleep quality became an even stronger independent predictor, and the number of completed modules showed a more apparent trend toward benefit (Supplementary Tables S1–S2).

Clinical interpretation (illustrative): Using the regression model, the predicted probability of response for a representative patient with average covariates changed from 25.4% to 22.3% with a one-point higher BFI, and from 25.4% to 27.7% with a one-unit higher sleep score. These scenarios are descriptive predictions, not causal effects, and are provided to aid clinical readability.

This study has several limitations. First, the retrospective design limits causal inference. A key limitation is the absence of pharmacological treatment data and treatment changes during follow-up. Therefore, our models may partially capture unmeasured treatment effects (confounding by indication) despite adjustment for proxies of baseline disease burden. Accordingly, associations should not be interpreted causally, and the analysis cannot disentangle app-supported self-management from concurrent medical care. Second, although we harmonised disease activity across diagnostic entities and adjusted all models for baseline disease-activity category, residual confounding by disease severity cannot be excluded. Sensitivity analyses restricted to patients in remission or low disease activity supported the robustness of the main findings but were limited by reduced sample size. Third, although PGADA and PPAIN were used as validated surrogates for composite scores (with strong correlations, r > 0.7), the absence of joint counts or CRP precluded the definition of complete clinical remission. Due to the strong correlation between PGADA and Pain VAS, only baseline Pain VAS was included in the primary model to avoid multicollinearity. Sleep and diet quality were assessed using app-specific composite indices without external validation against reference instruments, which limits interpretability and warrants cautious inference. Fourth, the cohort consisted of self-selected users of a German-language digital health app, who may differ from the broader IA population in motivation, digital literacy, and health behaviors, potentially limiting generalisability to non-digital populations. Moreover, follow-up availability may introduce selection related to retention and data completeness. Finally, the modest predictive accuracy (AUC ~ 0.62) underscores the complexity of pain prediction and the likely contribution of unmeasured factors, and because single imputation may influence nonlinear explainability outputs, any apparent threshold effects in SHAP analyses should be interpreted cautiously, considered hypothesis-generating, and require external validation before clinical use.

## Conclusions

This analysis identifies distinct multimodal predictors of short-term pain relief in IA. While high baseline pain mathematically favours a response, high fatigue appears to be a major barrier, and better sleep may be a resilience factor. The observation that SpA patients may be more responsive than PsA patients supports the need for tailored, phenotype-specific management strategies. At present, however, the models are intended for hypothesis generation rather than clinical decision support; external validation and incorporation of medication and treatment-change data are necessary before any individualized prediction or guidance could be considered. These findings nevertheless suggest that future digital therapeutics should move beyond “one-size-fits-all” modules and prospectively test more targeted fatigue- and sleep-focused support pathways in at-risk patients.

## Supplementary Information

Below is the link to the electronic supplementary material.

Supplementary file1 (DOCX 32 KB)

Supplementary file2 (DOCX 39 KB)

Supplementary file3 (DOCX 38 KB)

Supplementary file4 (DOCX 3202 KB)

Supplementary file5 (DOCX 2493 KB)

## Data Availability

Analysis was conducted on anonymized data, under a data-processing agreement, which allowed the authors to maintain independent analytic control. Data Sharing: Midaia GmbH data is not approved for sharing.
