# Supplementary material for: Baseline pain, fatigue, and sleep quality predict 12-week pain improvement in inflammatory arthritis: retrospective real-world analysis of a digital health application cohort
Source: Rheumatol Int. 2026 Apr 10;46(5):77. doi: 10.1007/s00296-026-06105-4 (PMC13068763; doi:10.1007/s00296-026-06105-4)
Supplement: Supplementary file 1 — Supplementary file1 (DOCX 32 KB) [file 296_2026_6105_MOESM1_ESM.docx]

**Appendix S1. STROBE Checklist (Cohort studies)**

Please indicate where each STROBE item is addressed in the manuscript (page/line or section). If not applicable, write “N/A”.

| **Section/Topic** | **Item** | **Recommendation** | **Reported on (page/line or section)** | **Notes (optional)** |
| --- | --- | --- | --- | --- |
| Title and abstract | 1a | Indicate the study’s design with a commonly used term in the title or the abstract | Title |  |
| Title and abstract | 1b | Provide in the abstract an informative and balanced summary of what was done and what was found | Abstract |  |
| Introduction | 2 | Explain the scientific background and rationale for the investigation being reported | Introduction |  |
| Introduction | 3 | State specific objectives, including any prespecified hypotheses | Introduction (final paragraph: study aim) |  |
| Methods | 4 | Present key elements of study design early in the paper | Methods - Study design and patients |  |
| Methods | 5 | Describe the setting, locations, and relevant dates, including periods of recruitment, exposure, follow-up, and data collection | Methods - Study design and patients (setting and dates: Jan 2022-Jun 2025) |  |
| Methods | 6a | Give the eligibility criteria, and the sources and methods of selection of participants. Describe methods of follow-up | Methods - Study design and patients (eligibility; analytical cohort N=914; follow-up at 12 weeks) |  |
| Methods | 6b | For matched studies, give matching criteria and number of exposed and unexposed | Not applicable (no matching) |  |
| Methods | 7 | Clearly define all outcomes, exposures, predictors, potential confounders, and effect modifiers. Give diagnostic criteria, if applicable | Methods - Assessments and Outcome Definition (predictors and outcome definition) |  |
| Methods | 8 | For each variable of interest, give sources of data and details of methods of assessment (measurement). Describe comparability of assessment methods if there is more than one group | Methods - Study design and patients; Methods - Assessments and Outcome Definition (measures/scales) |  |
| Methods | 9 | Describe any efforts to address potential sources of bias | Methods - Assessments and Outcome Definition (Sensitivity analysis only (baseline PPAIN ≥30 mm); primary analysis included all baseline pain levels); Discussion - Limitations (residual confounding) |  |
| Methods | 10 | Explain how the study size was arrived at | Methods - Study design and patients (N=2,924; prediction cohort N=914) |  |
| Methods | 11 | Explain how quantitative variables were handled in the analyses. If applicable, describe which groupings were chosen and why | Methods - Assessments and Outcome Definition (scales); Methods - Statistical Analysis (standardization; OR per 1 SD; units) |  |
| Methods | 12a | Describe all statistical methods, including those used to control for confounding | Methods - Statistical Analysis (adjusted models; confounding control) |  |
| Methods | 12b | Describe any methods used to examine subgroups and interactions | Methods - Statistical Analysis (sensitivity/subgroup analyses); Results (disease-activity adjusted and remission/low subgroup) |  |
| Methods | 12c | Explain how missing data were addressed | Methods - Statistical Analysis (single imputation: median/mode; exclusion rules) |  |
| Methods | 12d | If applicable, explain how loss to follow-up was addressed | Results (cohort derivation: 2,924 to 914 with week-12 pain) |  |
| Methods | 12e | Describe any sensitivity analyses | Methods - Statistical Analysis (sensitivity analyses); Results (baseline pain excluded; disease activity adjustment; remission/low subgroup) |  |
| Results | 13a | Report numbers of individuals at each stage of study (eg, numbers potentially eligible, examined for eligibility, confirmed eligible, included, completing follow-up, and analysed) | Results (participant flow: 2,924 registered users to 914 with week-12 pain) |  |
| Results | 13b | Give reasons for non-participation at each stage | Not fully reported (main reason stated as missing week-12 pain data) |  |
| Results | 13c | Consider use of a flow diagram | Not reported (no flow diagram) |  |
| Results | 14a | Give characteristics of study participants (eg, demographic, clinical, social) and information on exposures and potential confounders | Results - Table 1 (baseline characteristics by response status) |  |
| Results | 14b | Indicate number of participants with missing data for each variable of interest | Not reported (no per-predictor missingness table in main manuscript) |  |
| Results | 14c | Summarise follow-up time (eg, average and total amount) | Methods - Outcome Definition (fixed 12-week follow-up) |  |
| Results | 15 | Report numbers of outcome events or summary measures over time | Results (response rate 25.4%; baseline differences; Tables 1-2) |  |
| Results | 16a | Give unadjusted estimates and, if applicable, confounder-adjusted estimates and their precision (eg, 95% confidence interval). Make clear which confounders were adjusted for and why they were included | Results - Table 2; Figures 1-3 (adjusted ORs; regression coefficients; SHAP) |  |
| Results | 16b | Report category boundaries when continuous variables were categorized | Methods - Assessments and Outcome Definition (cut-offs; BFI/PHQ-4 categories; outcome threshold >=30%); Methods - Statistical Analysis (OR per 1 SD) |  |
| Results | 16c | If relevant, consider translating estimates of relative risk into absolute risk for a meaningful time period | Not applicable (absolute risk translation not presented) |  |
| Results | 17 | Report other analyses done (eg, analyses of subgroups and interactions, and sensitivity analyses) | Results (SHAP analyses; sensitivity analyses; subgroup analyses; Supplementary figures/tables referenced) |  |
| Discussion | 18 | Summarise key results with reference to study objectives | Discussion (opening: main findings) |  |
| Discussion | 19 | Discuss limitations of the study, taking into account sources of potential bias or imprecision. Discuss both direction and magnitude of any potential bias | Discussion (Limitations) |  |
| Discussion | 20 | Give a cautious overall interpretation of results considering objectives, limitations, multiplicity of analyses, results from similar studies, and other relevant evidence | Discussion (Interpretation; implications; comparison with literature) |  |
| Discussion | 21 | Discuss the generalisability (external validity) of the study results | Discussion (Generalisability limitation: self-selected app users) |  |
| Other information | 22 | Give the source of funding and the role of the funders for the present study and, if applicable, for the original study on which the present article is based | Funding/role of sponsor; Data access and availability |  |

*Source: STROBE Statement checklist for cohort studies.*
