# Supplementary material for: Baseline pain, fatigue, and sleep quality predict 12-week pain improvement in inflammatory arthritis: retrospective real-world analysis of a digital health application cohort
Source: Rheumatol Int. 2026 Apr 10;46(5):77. doi: 10.1007/s00296-026-06105-4 (PMC13068763; doi:10.1007/s00296-026-06105-4)
Supplement: Supplementary file 2 — Supplementary file2 (DOCX 39 KB) [file 296_2026_6105_MOESM2_ESM.docx]

**Appendix S2. TRIPOD Checklist (Prediction model development and validation)**

Please indicate where each TRIPOD item is addressed in the manuscript (page/line or section). If not applicable, write “N/A”.

| **Section/Topic** | **Item** | **D/V** | **Checklist item** | **Reported on (page/line or section)** | **Notes (optional)** |
| --- | --- | --- | --- | --- | --- |
| Title and abstract | 1 | D;V | Identify the study as developing and/or validating a multivariable prediction model, the target population, and the outcome to be predicted. | Title |  |
| Title and abstract | 2 | D;V | Provide a summary of objectives, study design, setting, participants, sample size, predictors, outcome, statistical analysis, results, and conclusions. | Abstract |  |
| Introduction - Background and objectives | 3a | D;V | Explain the medical context (including whether diagnostic or prognostic) and rationale for developing or validating the multivariable prediction model, including references to existing models. | Introduction (background and rationale) |  |
| Introduction - Background and objectives | 3b | D;V | Specify the objectives, including whether the study describes the development or validation of the model or both. | Introduction (objective paragraph) |  |
| Methods - Source of data | 4a | D;V | Describe the study design or source of data (eg, randomised trial, cohort, or registry data), separately for the development and validation datasets, if applicable. | Methods - Study design and patients (data source: app cohort; retrospective observational) |  |
| Methods - Source of data | 4b | D;V | Specify the key study dates, including start of accrual; end of accrual; and, if applicable, end of follow-up. | Methods - Study design and patients (dates: Jan 2022-Jun 2025; follow-up 12 weeks) |  |
| Methods - Participants | 5a | D;V | Specify key elements of the study setting (eg, primary care, secondary care, general population) including number and location of centres. | Methods - Study design and patients (setting; Germany; routine-use app cohort) |  |
| Methods - Participants | 5b | D;V | Describe eligibility criteria for participants. | Methods - Study design and patients (eligibility criteria; analytical cohort definition) |  |
| Methods - Participants | 5c | D;V | Give details of treatments received, if relevant. | Methods - Assessments and Outcome Definition (medication data not available); Discussion - Limitations |  |
| Methods - Outcome | 6a | D;V | Clearly define the outcome that is predicted by the prediction model, including how and when assessed. | Methods - Assessments and Outcome Definition (outcome: >=30% pain reduction at 12 weeks; Pain VAS) |  |
| Methods - Outcome | 6b | D;V | Report any actions to blind assessment of the outcome to be predicted. | Not applicable (self-reported outcome via app; no assessor blinding described) |  |
| Methods - Predictors | 7a | D;V | Clearly define all predictors used in developing or validating the multivariable prediction model, including how and when they were measured. | Methods - Assessments and Outcome Definition (predictor definitions and scoring) |  |
| Methods - Predictors | 7b | D;V | Report any actions to blind assessment of predictors for the outcome and other predictors. | Not applicable (predictors self-reported/baseline; no blinding described) |  |
| Methods - Sample size | 8 | D;V | Explain how the study size was arrived at. | Methods - Study design and patients (sample size N=914; source cohort N=2,924) |  |
| Methods - Missing data | 9 | D;V | Describe how missing data were handled (eg, complete-case analysis, single imputation, multiple imputation) with details of any imputation method. | Methods - Statistical Analysis (missing data handling; single imputation) |  |
| Methods - Statistical analysis methods | 10a | D | Describe how predictors were handled in the analyses. | Methods - Statistical Analysis (modeling approaches; covariate adjustment; cross-validation) |  |
| Methods - Statistical analysis methods | 10b | D | Specify type of model, all model-building procedures (including any predictor selection), and method for internal validation. | Methods - Statistical Analysis (model specification; predictors; scaling; Random Forest settings; class weights) |  |
| Methods - Statistical analysis methods | 10c | V | For validation, describe how the predictions were calculated. | Methods - Statistical Analysis (Repeated Stratified 5x2 cross-validation) |  |
| Methods - Statistical analysis methods | 10d | D;V | Specify all measures used to assess model performance and, if relevant, to compare multiple models. | Methods - Statistical Analysis (ROC AUC, sensitivity); Results (AUC ~0.61; R^2 for regressor) |  |
| Methods - Statistical analysis methods | 10e | V | Describe any model updating (eg, recalibration) arising from the validation, if done. | Not applicable (risk groups not created) |  |
| Methods - Risk groups | 11 | D;V | Provide details on how risk groups were created, if done. | Not applicable (no point score/nomogram provided) |  |
| Methods - Development vs validation | 12 | V | For validation, identify any differences from the development data in setting, eligibility criteria, outcome, and predictors. | Methods - Statistical Analysis (development with internal validation only; no external validation) |  |
| Results - Participants | 13a | D;V | Describe the flow of participants through the study, including the number of participants with and without the outcome and, if applicable, a summary of the follow-up time. A diagram may be helpful. | Results (flow: 2,924 to 914; responders 25.4%) |  |
| Results - Participants | 13b | D;V | Describe the characteristics of the participants (basic demographics, clinical features, available predictors), including the number of participants with missing data for predictors and outcome. | Results - Table 1 |  |
| Results - Participants | 13c | V | For validation, show a comparison with the development data of the distribution of important variables (demographics, predictors, and outcome). | Results (N=914; responders n=232); Results - Table 1 |  |
| Results - Model development | 14a | D | Specify the number of participants and outcome events in each analysis. | Results (N and events per analysis; response prevalence); Results - Table 1 and Table 2 |  |
| Results - Model development | 14b | D | If done, report the unadjusted association between each candidate predictor and outcome. | Results - Table 1 (unadjusted comparisons with p-values) |  |
| Results - Model specification | 15a | D | Present the full prediction model to allow predictions for individuals (eg, all regression coefficients, and model intercept or baseline survival at a given time point). | Not reported (full model specification for individual prediction not provided; Table 2 reports adjusted ORs without full equation) |  |
| Results - Model specification | 15b | D | Explain how to use the prediction model. | Not applicable (model not presented for clinical deployment) |  |
| Results - Model performance | 16 | D;V | Report performance measures (with confidence intervals) for the prediction model. | Results (model performance reported as AUC ~0.61; confidence intervals for AUC not reported) |  |
| Results - Model updating | 17 | V | If done, report the results from any model updating (eg, model specification, model performance). | Not applicable (no model updating) |  |
| Discussion - Limitations | 18 | D;V | Discuss any limitations of the study (such as non-representative sample, few events per predictor, missing data). | Discussion (Limitations) |  |
| Discussion - Interpretation | 19a | V | For validation, discuss the results with reference to performance in the development data, and any other validation data. | Not applicable (no external validation dataset) |  |
| Discussion - Interpretation | 19b | D;V | Give an overall interpretation of the results, considering objectives, limitations, results from similar studies, and other relevant evidence. | Discussion (overall interpretation; cautious, hypothesis-generating) |  |
| Discussion - Implications | 20 | D;V | Discuss the potential clinical use of the model and implications for future research. | Discussion (Clinical implications and future research; conclusion) |  |
| Other information - Supplementary information | 21 | D;V | Provide information about the availability of supplementary resources, such as study protocol, Web calculator, and datasets. | Data access and availability; Supplementary figures/tables referenced in text |  |
| Other information - Funding | 22 | D;V | Give the source of funding and the role of the funders for the present study. | Funding/role of sponsor; Conflict of interest |  |

** Items relevant only to the development of a prediction model are denoted by D; items relating solely to a validation of a prediction model are denoted by V; items relating to both are denoted D;V.*

*Source: TRIPOD checklist (2015) for prediction model development and validation.*
