# Supplementary material for: Baseline pain, fatigue, and sleep quality predict 12-week pain improvement in inflammatory arthritis: retrospective real-world analysis of a digital health application cohort
Source: Rheumatol Int. 2026 Apr 10;46(5):77. doi: 10.1007/s00296-026-06105-4 (PMC13068763; doi:10.1007/s00296-026-06105-4)
Supplement: Supplementary file 3 — Supplementary file3 (DOCX 38 KB) [file 296_2026_6105_MOESM3_ESM.docx]

Appendix S3. TRIPOD+AI Checklist (Prediction model studies using regression or machine learning)

Please indicate where each TRIPOD+AI item is addressed in the manuscript (page/line or section). If not applicable, write “N/A”.

| **Section/Topic** | **Item** | **D/E** | **Checklist item** | **Reported on (page/line or section)** | **Notes (optional)** |
| --- | --- | --- | --- | --- | --- |
| Title | 1 | D;E | Identify the study as developing or evaluating the performance of a multivariable prediction model, the target population, and the outcome to be predicted | Title |  |
| Abstract | 2 | D;E | See TRIPOD+AI for Abstracts checklist | Abstract (see AI abstracts checklist in Appendix S3) |  |
| Introduction - Background | 3a | D;E | Explain the healthcare context (including whether diagnostic or prognostic) and rationale for developing or evaluating the prediction model, including references to existing models | Introduction (background, rationale, related work) |  |
| Introduction - Background | 3b | D;E | Describe the target population and the intended purpose of the prediction model in the context of the care pathway, including its intended users (eg, healthcare professionals, patients, public) | Introduction; Methods - Study design and patients (digital self-management context) |  |
| Introduction - Background | 3c | D;E | Describe any known health inequalities between sociodemographic groups | Not reported (health inequalities/fairness not discussed) |  |
| Introduction - Objectives | 4 | D;E | Specify the study objectives, including whether the study describes the development or validation of a prediction model (or both) | Introduction (objective paragraph) |  |
| Methods - Data | 5a | D;E | Describe the sources of data separately for the development and evaluation datasets (eg, randomised trial, cohort, routine care or registry data), the rationale for using these data, and representativeness of the data | Methods - Study design and patients (data source); Discussion - Limitations (representativeness) |  |
| Methods - Data | 5b | D;E | Specify the dates of the collected participant data, including start and end of participant accrual; and, if applicable, end of follow-up | Methods - Study design and patients (dates: Jan 2022-Jun 2025; outcome horizon 12 weeks) |  |
| Methods - Participants | 6a | D;E | Specify key elements of the study setting (eg, primary care, secondary care, general population) including the number and location of centres | Methods - Study design and patients (setting; app users; Germany) |  |
| Methods - Participants | 6b | D;E | Describe the eligibility criteria for study participants | Methods - Study design and patients (eligibility; analytical cohort definition) |  |
| Methods - Participants | 6c | D;E | Give details of any treatments received, and how they were handled during model development or evaluation, if relevant | Methods - Assessments and Outcome Definition (medication data unavailable); Discussion - Limitations |  |
| Methods - Data preparation | 7 | D;E | Describe any data pre-processing and quality checking, including whether this was similar across relevant sociodemographic groups | Methods - Statistical Analysis (preprocessing and imputation); no formal data quality checks across sociodemographic groups reported |  |
| Methods - Outcome | 8a | D;E | Clearly define the outcome that is being predicted and the time horizon, including how and when assessed, the rationale for choosing this outcome, and whether the method of outcome assessment is consistent across sociodemographic groups | Methods - Assessments and Outcome Definition (outcome definition; 12-week horizon; >=30% reduction) |  |
| Methods - Outcome | 8b | D;E | If outcome assessment requires subjective interpretation, describe the qualifications and demographic characteristics of the outcome assessors | Not applicable (self-reported outcome; no assessor interpretation) |  |
| Methods - Outcome | 8c | D;E | Report any actions to blind assessment of the outcome to be predicted | Not applicable (self-reported outcome; no blinding described) |  |
| Methods - Predictors | 9a | D | Describe the choice of initial predictors (eg, literature, previous models, all available predictors) and any pre-selection of predictors before model building | Methods - Assessments and Outcome Definition (candidate predictors specified); Introduction (behavioral/lifestyle predictors rationale) |  |
| Methods - Predictors | 9b | D;E | Clearly define all predictors, including how and when they were measured (and any actions to blind assessment of predictors for the outcome and other predictors) | Methods - Assessments and Outcome Definition (predictor definitions and timing at baseline); Methods - Statistical Analysis (scaling/standardization) |  |
| Methods - Predictors | 9c | D;E | If predictor measurement requires subjective interpretation, describe the qualifications and demographic characteristics of the predictor assessors | Not applicable (predictors are self-reported; no assessor interpretation) |  |
| Methods - Sample size | 10 | D;E | Explain how the study size was arrived at (separately for development and evaluation), and justify that the study size was sufficient to answer the research question. Include details of any sample size calculation | Methods - Study design and patients (sample size N=914; cohort derivation); no formal sample size calculation reported |  |
| Methods - Missing data | 11 | D;E | Describe how missing data were handled. Provide reasons for omitting any data | Methods - Statistical Analysis (single imputation; exclusion rules for high missingness) |  |
| Methods - Analytical methods | 12a | D | Describe how the data were used (eg, for development and evaluation of model performance) in the analysis, including whether the data were partitioned, considering any sample size requirements | Methods - Statistical Analysis (development with internal validation; regression + Random Forest + SHAP) |  |
| Methods - Analytical methods | 12b | D | Depending on the type of model, describe how predictors were handled in the analyses (functional form, rescaling, transformation, or any standardisation) | Methods - Statistical Analysis (standardization; functional form; OR per 1 SD; predictors in original units for linear regression) |  |
| Methods - Analytical methods | 12c | D | Specify the type of model, rationale, all model building steps, including any hyperparameter tuning, and method for internal validation | Methods - Statistical Analysis (model types; Random Forest settings; class weights; cross-validation; software versions) |  |
| Methods - Analytical methods | 12d | D;E | Describe if and how any heterogeneity in estimates of model parameter values and model performance was handled and quantified across clusters (eg, hospitals, countries). See TRIPOD-Cluster for additional considerations | Methods - Statistical Analysis (disease-activity adjusted sensitivity and remission/low subgroup); Results (sensitivity/subgroup findings) |  |
| Methods - Analytical methods | 12e | D;E | Specify all measures and plots used (and their rationale) to evaluate model performance (eg, discrimination, calibration, clinical utility) and, if relevant, to compare multiple models | Methods - Statistical Analysis (ROC AUC, sensitivity, R^2; SHAP plots); Results (performance reporting; Figures/Supplementary) |  |
| Methods - Analytical methods | 12f | E | Describe any model updating (eg, recalibration) arising from the model evaluation, either overall or for particular sociodemographic groups or settings | Not applicable (no model updating/recalibration) |  |
| Methods - Analytical methods | 12g | E | For model evaluation, describe how the model predictions were calculated (eg, formula, code, object, application programming interface) | Methods - Statistical Analysis (Random Forest classifier probabilities via scikit-learn; logistic regression for ORs); full prediction formula not provided |  |
| Methods - Class imbalance | 13 | D;E | If class imbalance methods were used, state why and how this was done, and any subsequent methods to recalibrate the model or the model predictions | Methods - Statistical Analysis (class imbalance: inverse-frequency class weights) |  |
| Methods - Fairness | 14 | D;E | Describe any approaches that were used to address model fairness and their rationale | Not reported (no explicit fairness assessment/mitigation methods) |  |
| Methods - Model output | 15 | D | Specify the output of the prediction model (eg, probabilities, classification). Provide details and rationale for any classification and how the thresholds were identified | Methods - Statistical Analysis (binary response classification; predicted probability output implied); Results (AUC reported; no probability threshold specified) |  |
| Methods - Training versus evaluation | 16 | D;E | Identify any differences between the development and evaluation data in healthcare setting, eligibility criteria, outcome, and predictors | Not applicable (no separate external evaluation dataset; internal cross-validation only) |  |
| Methods - Ethical approval | 17 | D;E | Name the institutional research board or ethics committee that approved the study and describe the participant informed consent or the ethics committee waiver of informed consent | Methods - Study design and patients (ethics/consent statement); ETHICAL APPROVAL; INFORMED CONSENT |  |
| Open science - Funding | 18a | D;E | Give the source of funding and the role of the funders for the present study | Funding/role of sponsor |  |
| Open science - Conflicts of interest | 18b | D;E | Declare any conflicts of interest and financial disclosures for all authors | CONFLICT OF INTEREST |  |
| Open science - Protocol | 18c | D;E | Indicate where the study protocol can be accessed or state that a protocol was not prepared | Not reported (protocol access not provided) |  |
| Open science - Registration | 18d | D;E | Provide registration information for the study, including register name and registration number, or state that the study was not registered | Not reported (study registration not provided) |  |
| Open science - Data sharing | 18e | D;E | Provide details of the availability of the study data | DATA ACCESS AND AVAILABILITY |  |
| Open science - Code sharing | 18f | D;E | Provide details of the availability of the analytical code | Not reported (analytical code availability not stated) |  |
| Patient and public involvement | 19 | D;E | Provide details of any patient and public involvement during the design, conduct, reporting, interpretation, or dissemination of the study or state no involvement | Not reported (patient/public involvement not described) |  |
| Results - Participants | 20a | D;E | Describe the flow of participants through the study, including the number of participants with and without the outcome and, if applicable, a summary of the follow-up time. A diagram may be helpful | Results (participant flow: 2,924 to 914 with week-12 pain; responders 25.4%); Table 1 |  |
| Results - Participants | 20b | D;E | Report the characteristics overall and, where applicable, for each data source or setting, including the key dates, key predictors (including demographics), treatments received, sample size, number of outcome events, follow-up time, and amount of missing data. A table may be helpful. Report any differences across key demographic groups | Results - Table 1 (baseline characteristics); Methods - Study design and patients (key dates/setting) |  |
| Results - Participants | 20c | E | For model evaluation, show a comparison with the development data of the distribution of important predictors (demographics, predictors, and outcome) | Not applicable (no separate development vs evaluation dataset; internal cross-validation only) |  |
| Results - Model development | 21 | D;E | Specify the number of participants and outcome events in each analysis (eg, for model development, hyperparameter tuning, model evaluation) | Results (N=914; responders n=232; response prevalence 25.4%); Table 1; Table 2 |  |
| Results - Model specification | 22 | D | Provide details of the full prediction model (eg, formula, code, object, application programming interface) to allow predictions in new individuals and to enable third party evaluation and implementation, including any restrictions to access or reuse (eg, freely available, proprietary) | Not reported (full model specification for individual prediction not provided) |  |
| Results - Model performance | 23a | D;E | Report model performance estimates with confidence intervals, including for any key subgroups (eg, sociodemographic). Consider plots to aid presentation | Results (AUC ~0.61; R^2 ~0.50; OR/β with 95% CIs in Table 2); AUC confidence intervals not reported |  |
| Results - Model performance | 23b | D;E | If examined, report results of any heterogeneity in model performance across clusters. See TRIPOD-Cluster for additional details | Not applicable (no cluster-heterogeneity analyses reported) |  |
| Results - Model updating | 24 | E | Report the results from any model updating, including the updated model and subsequent performance | Not applicable (no model updating) |  |
| Discussion - Interpretation | 25 | D;E | Give an overall interpretation of the main results, including issues of fairness in the context of the objectives and previous studies | Discussion (overall interpretation; hypothesis-generating; fairness not addressed) |  |
| Discussion - Limitations | 26 | D;E | Discuss any limitations of the study (such as a non-representative sample, sample size, overfitting, missing data) and their effects on any biases, statistical uncertainty, and generalisability | Discussion (Limitations; generalisability; missing medication data; modest discrimination) |  |
| Discussion - Usability | 27a | D | Describe how poor quality or unavailable input data (eg, predictor values) should be assessed and handled when implementing the prediction model | Not applicable (implementation guidance not provided; model not intended for deployment) |  |
| Discussion - Usability | 27b | D | Specify whether users will be required to interact in the handling of the input data or use of the model, and what level of expertise is required of users | Not applicable (model not intended for deployment; no user interaction requirements specified) |  |
| Discussion - Usability | 27c | D;E | Discuss any next steps for future research, with a specific view to applicability and generalisability of the model | Discussion (Conclusion and future research: external validation; richer treatment data) |  |

**TRIPOD+AI for Abstracts checklist**

Complete this table if the journal requests the abstracts checklist separately.

| **Section/Item** | **Checklist item** | **Reported on (page/line or section)** | **Notes (optional)** |
| --- | --- | --- | --- |
| Title - 1 | Identify the study as developing or evaluating the performance of a multivariable prediction model, the target population, and the outcome to be predicted | Abstract (opening sentence: design/target/outcome) |  |
| Background - 2 | Provide a brief explanation of the healthcare context and rationale for developing or evaluating the performance of all models | Abstract (Background) |  |
| Objectives - 3 | Specify the study objectives, including whether the study describes model development, evaluation, or both | Abstract (Objectives) |  |
| Methods - 4 | Describe the sources of data | Abstract (Methods: data source/app cohort) |  |
| Methods - 5 | Describe the eligibility criteria and setting where the data were collected | Abstract (Methods: inclusion/setting) |  |
| Methods - 6 | Specify the outcome to be predicted by the model, including time horizon of predictions in case of prognostic models | Abstract (Methods: response definition; 12-week horizon) |  |
| Methods - 7 | Specify the type of model, a summary of the model-building steps, and the method for internal validation (development studies only) | Abstract (Methods: regression + Random Forest; internal CV) |  |
| Methods - 8 | Specify the measures used to assess model performance (eg, discrimination, calibration, clinical utility) | Abstract (Results: discrimination metrics; calibration not reported) |  |
| Results - 9 | Report the number of participants and outcome events | Abstract (Results: N=914; responders n=232) |  |
| Results - 10 | Summarise the predictors in the final model (development studies only) | Abstract (Results: key predictors) |  |
| Results - 11 | Report model performance estimates (with confidence intervals) | Abstract (Results: AUC and R^2; CIs not reported) |  |
| Discussion - 12 | Give an overall interpretation of the main results | Abstract (Conclusion: hypothesis-generating) |  |
| Registration - 13 | Give the registration number and name of the registry or repository | Not reported (study not registered) |  |

*Source: Collins GS, et al. TRIPOD+AI statement (BMJ 2024; available via PubMed Central).*
