# Supplementary material for: Baseline pain, fatigue, and sleep quality predict 12-week pain improvement in inflammatory arthritis: retrospective real-world analysis of a digital health application cohort
Source: Rheumatol Int. 2026 Apr 10;46(5):77. doi: 10.1007/s00296-026-06105-4 (PMC13068763; doi:10.1007/s00296-026-06105-4)
Supplement: Supplementary file 4 — Supplementary file4 (DOCX 3202 KB) [file 296_2026_6105_MOESM4_ESM.docx]

### Supplementary Appendix S4. Definition and Scoring of Engineered Indices

**1) Sleep Quality Score (range -4 to +4)**

The Sleep Quality Score is a composite metric that captures both the subjective assessment of sleep sufficiency and the burden of specific sleep disturbances. The index balances positive sleep perception against the presence of common insomnia symptoms and parasomnias.

Sleep parameters were assessed via two distinct user-reported components:

**Subjective Satisfaction:** users rated their overall sleep satisfaction on a 5-point Likert scale (0 = Very Dissatisfied to 4 = Very Satisfied).

**Sleep Disturbances:** Users reported the presence (binary: Yes/No) of four specific sleep complaints during the recall period:

1. *Difficulty initiating sleep* ("Can’t sleep")
2. *Sleep maintenance difficulty* ("Wakes up at night")
3. *Early morning awakening* ("Wakes up too early")
4. *Nightmares*

The final score was calculated by subtracting the cumulative burden of sleep disturbances from the satisfaction score:

Sleep Quality Score=Sleep Satisfaction (0–4)−∑(Sleep Problems)

Where:

**Sleep Satisfaction** is the raw Likert value (0–4).

**Sum (Sleep Problems)** is the count of reported disturbances (range 0–4), where each present symptom contributes 1 point to the penalty.

The resulting index ranges from **-4** (representing maximum dissatisfaction combined with all four sleep disturbances) to **+4** (representing maximum satisfaction with zero disturbances). Higher values indicate better overall sleep quality. A score of 0 represents a neutral balance between satisfaction and disturbance burden.

**2) Diet Quality Score (range 0–100)**

The Diet Quality Score is a composite index designed to quantify adherence to anti-inflammatory dietary patterns (e.g., Mediterranean-style diet). Higher scores indicate a nutritional profile rich in anti-inflammatory foods and low in pro-inflammatory constituents.

Dietary intake was assessed via a simplified Food Frequency Questionnaire (FFQ) embedded within the application. Users reported their average consumption frequency across eight distinct food groups selected for their relevance to inflammatory arthritis:

1. *Fruits*
2. *Vegetables*
3. *Oily fish (e.g., salmon, mackerel)*
4. *White fish*
5. *Dairy products*
6. *Meat (unprocessed red/white meat)*
7. *Processed meat*
8. *Sugar and confectionery*

For each food group, consumption frequency was recorded on a 5-point ordinal scale:

**1 = Never**

**2 = Rarely (e.g., once a month)**

**3 = Occasionally (e.g., once a week)**

**4 = Frequently (e.g., several times a week)**

**5 = Very Frequently (4 or more times per day/week, context-dependent)**

Raw frequency scores were transformed into a standardized 0–100 index using the app’s internal algorithm. This algorithm assigns positive weights to protective food groups (e.g., fruits, vegetables, oily fish) and negative weights to potentially pro-inflammatory groups (e.g., sugar, processed meat), summing the weighted components to produce the final continuous score.

This index serves as an app-specific proxy for dietary quality, intended for longitudinal self-monitoring. It has not yet been validated against "gold standard" nutritional assessment tools (e.g., the Harvard Semi-Quantitative FFQ or 24-hour recall interviews) within this specific cohort. Consequently, associations between the Diet Quality Score and clinical outcomes should be interpreted as exploratory signals of nutritional impact rather than definitive causal evidence.
