# Supplementary material for: Baseline pain, fatigue, and sleep quality predict 12-week pain improvement in inflammatory arthritis: retrospective real-world analysis of a digital health application cohort
Source: Rheumatol Int. 2026 Apr 10;46(5):77. doi: 10.1007/s00296-026-06105-4 (PMC13068763; doi:10.1007/s00296-026-06105-4)
Supplement: Supplementary file 5 — Supplementary file5 (DOCX 2493 KB) [file 296_2026_6105_MOESM5_ESM.docx]

**Supplementary Table S1. Multivariable prediction models adjusted for harmonised baseline disease activity (subset with complete data for disease-activity classification; N=562)**

| **Predictor** | **Binary Response (≥30% Improvement) Adjusted OR (95% CI)** | **p-value** | **Magnitude of Improvement (ΔPPAIN) β Coefficient (95% CI)** | **p-value** |
| --- | --- | --- | --- | --- |
| **Harmonised Baseline Activity** | | | | |
| Remission/Low (ref: High) | **2.36 (1.03 to 5.41)** | **0.043** | **8.71 (2.11 to 15.32)** | **0.010** |
| Moderate (ref: High) | 0.92 (0.52 to 1.63) | 0.775 | 1.48 (-3.05 to 6.01) | 0.521 |
| **Clinical Factors** | | | | |
| Baseline Pain Intensity (0–100) | **2.04 (1.49 to 2.78)** | **<0.001** | **16.31 (13.94 to 18.68)** | **<0.001** |
| Fatigue (BFI, 0–10) | 0.77 (0.58 to 1.02) | 0.069 | **-3.55 (-5.88 to -1.21)** | **0.003** |
| Disease Duration | 0.89 (0.71 to 1.12) | 0.320 | 0.28 (-1.49 to 2.04) | 0.758 |
| **Psychosocial & Lifestyle** | | | | |
| Sleep Quality Score | 1.23 (0.99 to 1.53) | 0.067 | **2.90 (1.10 to 4.69)** | **0.002** |
| Diet Quality Score | 1.11 (0.89 to 1.37) | 0.354 | 0.60 (-1.08 to 2.28) | 0.486 |
| Social Support Score | 0.96 (0.77 to 1.20) | 0.724 | -0.57 (-2.32 to 1.19) | 0.527 |
| Psychological Distress (PHQ-4) | 1.03 (0.81 to 1.30) | 0.838 | 0.38 (-1.58 to 2.33) | 0.704 |
| Smoking | 1.12 (0.92 to 1.36) | 0.273 | 0.37 (-1.28 to 2.02) | 0.658 |
| **App Engagement** | | | | |
| Modules Completed | 1.06 (0.86 to 1.32) | 0.579 | 0.41 (-1.37 to 2.20) | 0.649 |
| App Compliance | 0.95 (0.76 to 1.20) | 0.686 | 0.39 (-1.42 to 2.20) | 0.672 |
| **Demographics** | | | | |
| Age (years) | 1.11 (0.89 to 1.39) | 0.334 | -0.38 (-2.14 to 1.39) | 0.676 |
| Male Sex (ref: Female) | 1.37 (0.83 to 2.28) | 0.220 | 2.68 (-1.49 to 6.84) | 0.207 |
| BMI | 1.07 (0.88 to 1.31) | 0.493 | 0.07 (-1.58 to 1.73) | 0.931 |
| Diagnosis: RA (ref: PsA) | 1.47 (0.81 to 2.65) | 0.202 | 3.39 (-1.22 to 7.99) | 0.149 |
| Diagnosis: SpA (ref: PsA) | 1.10 (0.55 to 2.19) | 0.787 | -3.78 (-9.14 to 1.58) | 0.167 |

Results from logistic (predicting binary response ≥30%) and linear (predicting magnitude of improvement) regression models trained on the full analytical cohort (N=562) with complete data for disease activity classification. All models are adjusted for harmonised disease activity categories to control for confounding by inflammatory burden. Bold values indicate statistical significance (p<0.05). Adjusted Odds Ratios (OR) and Beta coefficients (β) for continuous predictors are standardized (calculated per 1 Standard Deviation increase) to allow for direct comparison of effect sizes.

Abbreviations: BFI, Brief Fatigue Inventory; PHQ-4, Patient Health Questionnaire-4; RA, Rheumatoid Arthritis; SpA, Spondyloarthritis; PsA, Psoriatic Arthritis.

**Supplementary Table S2. Sensitivity analysis: Predictors of pain relief in patients with Remission/Low Disease Activity**

| **Predictor** | **Binary Response (≥30% Improvement) Adjusted OR (95% CI)** | **p-value** | **Magnitude of Improvement (ΔPPAIN) β Coefficient (95% CI)** | **p-value** |
| --- | --- | --- | --- | --- |
| **Psychosocial & Lifestyle** | | | | |
| **Sleep Quality Score** | **2.08 (1.13 to 3.85)** | **0.019** | **6.06 (1.59 to 10.53)** | **0.008** |
| Diet Quality Score | 1.63 (0.87 to 3.07) | 0.128 | 1.71 (-2.54 to 5.95) | 0.427 |
| Social Support Score | 0.68 (0.35 to 1.32) | 0.256 | -4.44 (-9.15 to 0.26) | 0.064 |
| **App Engagement** | | | | |
| Modules Completed | 1.67 (0.94 to 2.96) | 0.079 | 2.36 (-2.08 to 6.80) | 0.293 |
| App Compliance | 0.62 (0.34 to 1.12) | 0.113 | -2.71 (-7.11 to 1.68) | 0.223 |
| **Clinical Factors** | | | | |
| Baseline Pain Intensity (0–100) | 1.67 (0.94 to 2.97) | 0.078 | **10.00 (5.69 to 14.31)** | **<0.001** |
| Fatigue (BFI) | 0.95 (0.48 to 1.87) | 0.881 | -3.89 (-8.73 to 0.96) | 0.114 |
| Disease Duration | 1.23 (0.71 to 2.15) | 0.460 | **4.57 (0.36 to 8.79)** | **0.034** |
| **Demographics** | | | | |
| Age (years) | 1.52 (0.79 to 2.93) | 0.213 | -0.30 (-4.89 to 4.29) | 0.896 |
| Male Sex (ref: Female) | 1.83 (0.49 to 6.79) | 0.364 | 3.02 (-7.13 to 13.18) | 0.556 |
| BMI | 1.17 (0.71 to 1.95) | 0.533 | -1.11 (-4.95 to 2.73) | 0.567 |
| Diagnosis: RA (ref: PsA) | 2.93 (0.24 to 36.23) | 0.402 | 3.46 (-12.17 to 19.09) | 0.661 |
| Diagnosis: SpA (ref: PsA) | 3.00 (0.24 to 37.21) | 0.393 | -0.85 (-16.49 to 14.79) | 0.915 |

Multivariable models restricted to the subgroup of patients classified as being in remission or low disease activity at baseline (N=108). This analysis assesses the robustness of behavioral predictors when the dominant influence of high systemic inflammation is minimized. Note that Sleep Quality is a stronger predictor in this subgroup than in the whole cohort. Bold values indicate statistical significance (p<0.05). Adjusted Odds Ratios (OR) and Beta coefficients (β) are standardized (per 1 SD increase).

Abbreviations: BFI, Brief Fatigue Inventory; PHQ-4, Patient Health Questionnaire-4; RA, Rheumatoid Arthritis; SpA, Spondyloarthritis; PsA, Psoriatic Arthritis.

**Supplementary Table S3. Missingness of candidate predictors in the predictive cohort (N = 914).**

| **Predictor (unit/scale)** | **Missing n (%)** | **Handling/notes** |
| --- | --- | --- |
| Age (years) | 0 (0.0%) | Not imputed |
| Sex (female/male) | 0 (0.0%) | Not imputed |
| Diagnosis (RA/PsA/SpA) | 0 (0.0%) | Not imputed |
| Disease duration (years) | 90 (9.8%) | Imputed (median) |
| Body mass index (kg/m²) | 8 (0.9%) | Calculated from self-reported height/weight; remaining missing values imputed (median) |
| Baseline pain (PPAIN, 0-100 mm VAS) | 0 (0.0%) | Not imputed |
| Patient global assessment (PGADA, 0-100) | 0 (0.0%) | Not imputed |
| Fatigue (BFI, 0-10) | 0 (0.0%) | Not imputed |
| Sleep quality score (app index, -4 to 4) | 1 (0.1%) | Computed per scoring algorithm, remaining missing values imputed (median) |
| Psychological distress (PHQ-4, 0-12) | 149 (16.3%) | Imputed (median) |
| Physical activity index (app indicator) | 1 (0.1%) | Computed per scoring algorithm, remaining missing values imputed (median) |
| Social support index (app indicator) | 5 (0.5%) | Computed per scoring algorithm, remaining missing values imputed (median) |
| Diet quality score (app index) | 4 (0.4%) | Imputed (median) |
| App compliance (12-week follow-up) | 239 (26.1%) | Imputed (median) |
| App modules completed (12-week follow-up) | 239 (26.1%) | Imputed (median) |

Notes: Missingness is reported prior to imputation. Variables with >30% missingness were generally excluded from modelling, except for engineered indices where missingness was informative or handled by the scoring algorithm (as specified in Methods).

**Supplementary Table S4. Baseline comparison of included participants (predictive cohort with week-12 pain) versus excluded eligible app users without week-12 pain**

| **Characteristic¹** | **Included (N=914)** | **Excluded (N=2010)** | **p-value²** |
| --- | --- | --- | --- |
| Demographics | | | |
| Age, years | 46.86 ± 12.78 | 44.28 ± 13.31 | < 0.001 |
| Male sex, n (%) | 182 (19.9%) | 481 (23.9%) | < 0.001 |
| Disease Duration, years | 8.58 ± 9.09 | 8.26 ± 9.66 | 0.434 |
| Body Mass Index, kg/m² | 26.83 ± 5.88 | 27.09 ± 6.18 | 0.311 |
| Smoking, packs | 1.86 ± 1.13 | 2.08 ± 1.19 | < 0.001 |
| Diagnosis, n (%) | | | |
| Rheumatoid Arthritis | 529 (57.9%) | 1278 (63.6%) | < 0.001 |
| Spondyloarthritis | 255 (27.9%) | 498 (24.8%) | < 0.001 |
| Psoriatic Arthritis | 130 (14.2%) | 234 (11.6%) | < 0.001 |
| Clinical Symptoms | | | |
| Pain Intensity (PPAIN), 0–100 | 51.62 ± 25.11 | 54.93 ± 25.76 | 0.002 |
| Disease Activity (PGADA), 0–100 | 56.63 ± 25.01 | 59.53 ± 25.48 | 0.005 |
| Fatigue (BFI), 0–10 | 4.98 ± 1.98 | 5.32 ± 1.93 | < 0.001 |
| Psychosocial & Lifestyle | | | |
| Psychological Distress (PHQ-4), 0–12 | 3.99 ± 2.60 | 4.20 ± 2.77 | 0.107 |
| Sleep Quality Score, -4 to +4 | 0.51 ± 1.72 | 0.40 ± 1.72 | 0.116 |
| Diet Quality Score, 0–100 | 69.92 ± 15.62 | 68.16 ± 15.92 | 0.009 |
| Social Support Score, 0–20 | 13.63 ± 4.18 | 13.18 ± 4.35 | 0.014 |
| Physical Activity Score, 0–20 | 7.02 ± 4.89 | 6.83 ± 4.64 | 0.348 |
| App Engagement | | | |
| App Compliance, % | 51.92 ± 23.56 | 56.10 ± 25.29 | 0.192 |
| Modules Completed, n | 5.65 ± 6.44 | 1.77 ± 1.63 | < 0.001 |
| ¹ Data are presented as mean ± standard deviation for continuous variables and n (%) for categorical variables. | | | |
| ² p-values were calculated using the Student’s t-test for continuous variables and Pearson’s Chi-squared test for categorical variables. | | | |

Notes:

Included participants are those in the predictive cohort with baseline pain and week-12 pain available; excluded participants are eligible app users without week-12 pain recorded.

**Figure S1. Concurrent validity of single-item Pain Intensity (PPAIN) against composite disease activity scores.** Scatter plots showing Spearman correlations in the sub-cohort of patients with complete data. **(A)** Strong correlation between PPAIN and RAPID3 in patients with RA/PsA (r=0.80, p<0.001). **(B)** Strong correlation between PPAIN and BASDAI in patients with SpA (r=0.72, p<0.001). This supports the pragmatic use of PPAIN as a surrogate measure of disease activity in this analysis.

**Abbreviations:** **PPAIN**, Patient Global Assessment of Pain Intensity; **RAPID3**, Routine Assessment of Patient Index Data 3; **RA**, Rheumatoid Arthritis; **PsA**, Psoriatic Arthritis; **SpA**, Spondyloarthritis; **BASDAI**, Bath Ankylosing Spondylitis Disease Activity Index.

**Figure S2. Calibration curve for the Random Forest classifier predicting ≥30% pain reduction over 12 weeks.** The plot compares the mean predicted probability of response (x-axis) against the actual observed fraction of responders (y-axis). The dashed diagonal line represents perfect calibration. The model shows acceptable calibration, indicating reliable probability estimates across risk groups.

**Figure S3. Decision Curve Analysis (DCA) for the Random Forest classifier predicting ≥30% pain reduction.** The graph illustrates the net benefit of using the prediction model (blue line) to guide clinical decision-making compared to default strategies of "Treat All" (grey line) or "Treat None" (black line). The model provides a positive net benefit across a wide range of threshold probabilities, supporting its potential clinical utility.

**Figure S4. Comparison of feature importance for all predictors in Random Forest classification and regression models.** The bar chart displays the relative importance (Mean Decrease in Impurity, %) of every predictor included in the machine learning analysis. Blue bars represent the Random Forest Classifier (predicting the likelihood of achieving ≥30% pain reduction), while orange bars represent the Random Forest Regressor (predicting the continuous magnitude of pain reduction). Notably, while Baseline Pain Intensity dominates the regression model (34.2%) due to the mathematical "headroom effect," Baseline Fatigue (BFI) emerges as the most important feature for classifying successful response (10.7%), surpassing pain intensity in the binary classification task. This highlights fatigue as a critical biological barrier to clinically meaningful improvement.

Abbreviations: BFI, Brief Fatigue Inventory; PHQ-4, Patient Health Questionnaire-4; BMI, Body Mass Index; RA, Rheumatoid Arthritis; SpA, Spondyloarthritis; PsA, Psoriatic Arthritis.

**Figure S5. Forest plot of adjusted Odds Ratios from the logistic regression model for sensitivity analysis (Full Cohort).** The model (N=562) is adjusted for harmonised baseline disease activity categories alongside all other covariates. The plot demonstrates that patients classified as being in the "Remission/Low activity" category at baseline have significantly higher odds of achieving a clinically meaningful pain reduction compared to the reference group ("High Activity") (OR 2.36), even after accounting for baseline pain intensity.

Abbreviations: BFI, Brief Fatigue Inventory; PHQ-4, Patient Health Questionnaire-4; RA, Rheumatoid Arthritis; SpA, Spondyloarthritis; PsA, Psoriatic Arthritis; OR, Odds Ratio; CI, Confidence Interval.

**Figure S6. Forest plot for the sensitivity analysis restricted to patients with Remission/Low Disease Activity.** This analysis, performed on a subgroup of patients (N=108), identifies predictors of pain response specifically in the context of low inflammatory burden. Sleep Quality emerges as the strongest significant predictor (OR 2.08), suggesting that sleep optimization may be a critical mechanism for symptom relief in this patient category. Confidence Intervals are wider than in the main model due to the reduced sample size.

Abbreviations: CI, Confidence Interval; OR, Odds Ratio.


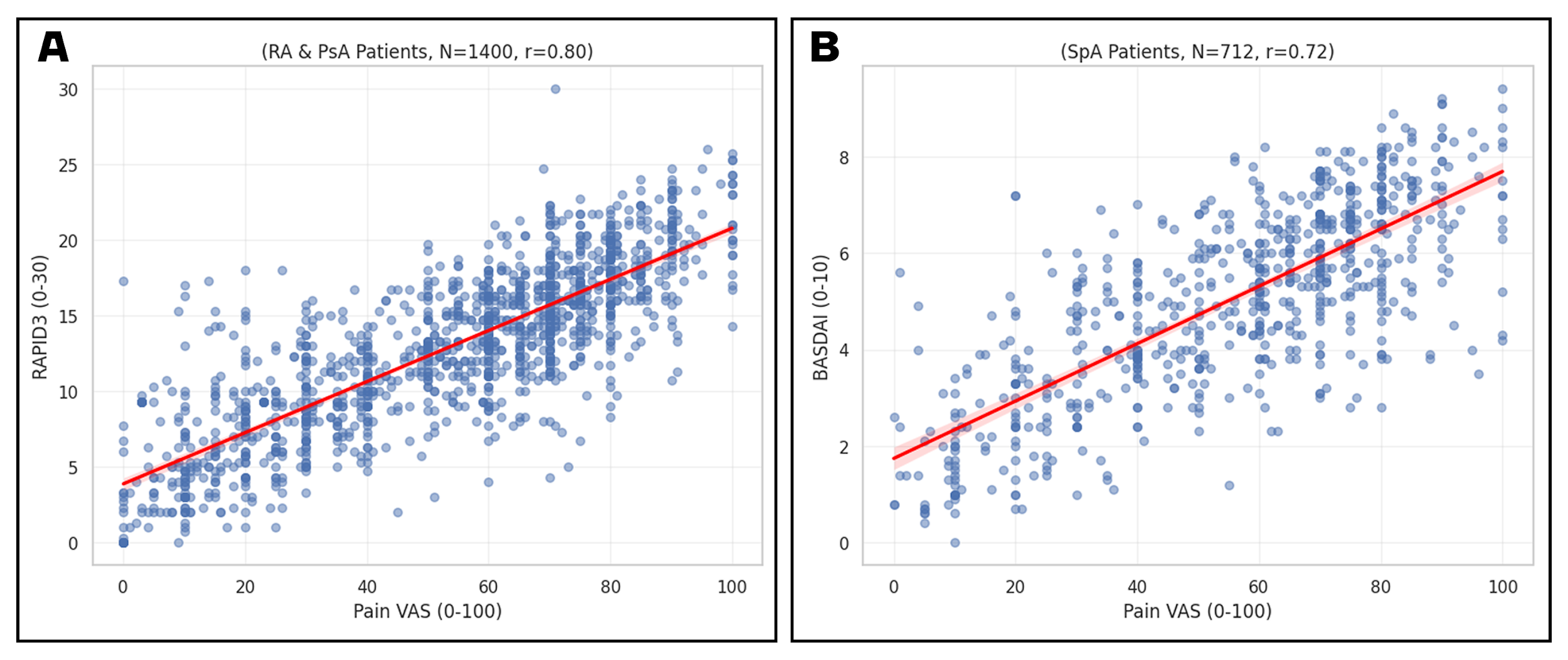
**Figure S**1


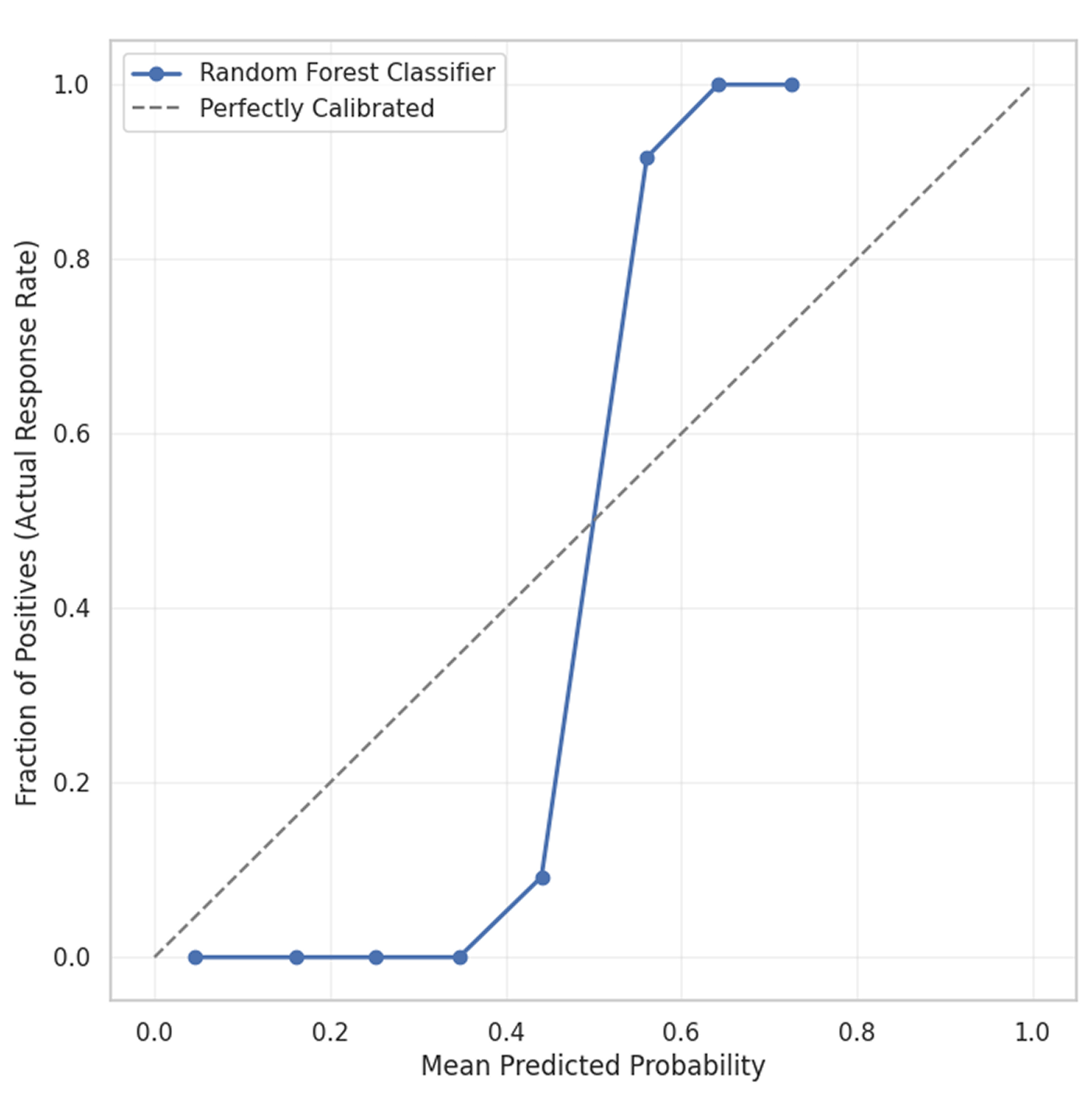
**Figure S**2

**Figur**
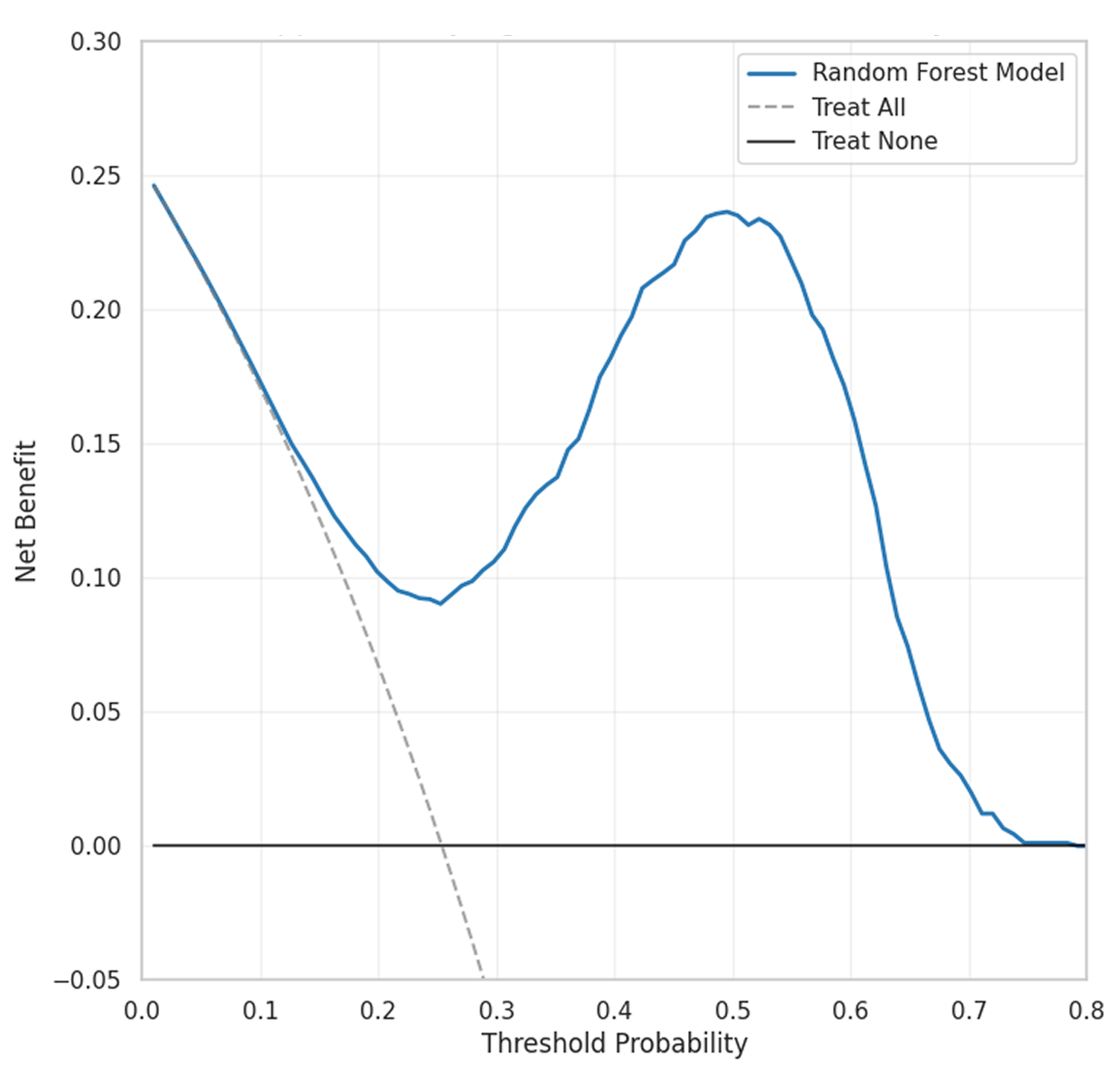
**e S3**


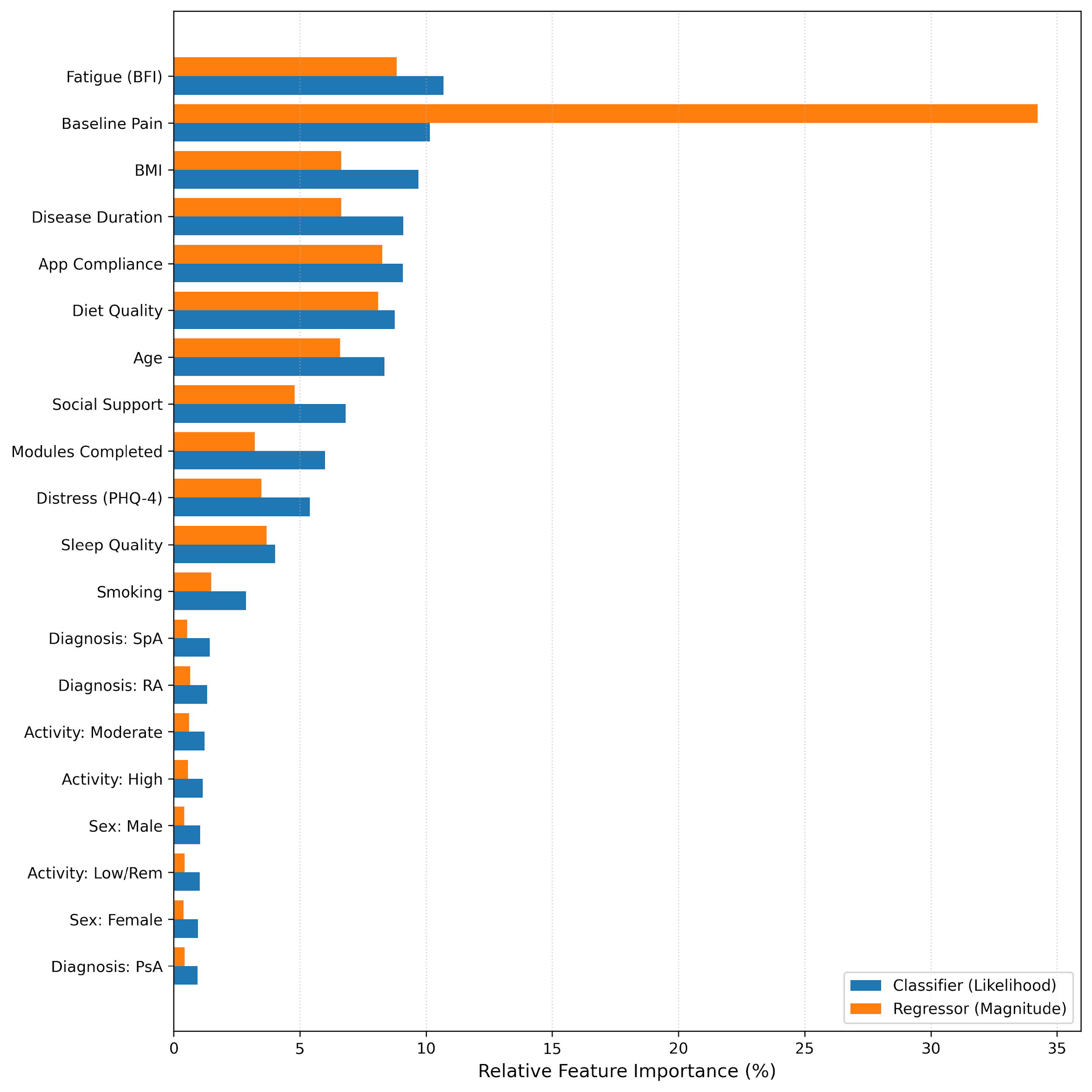
**Figure S**4


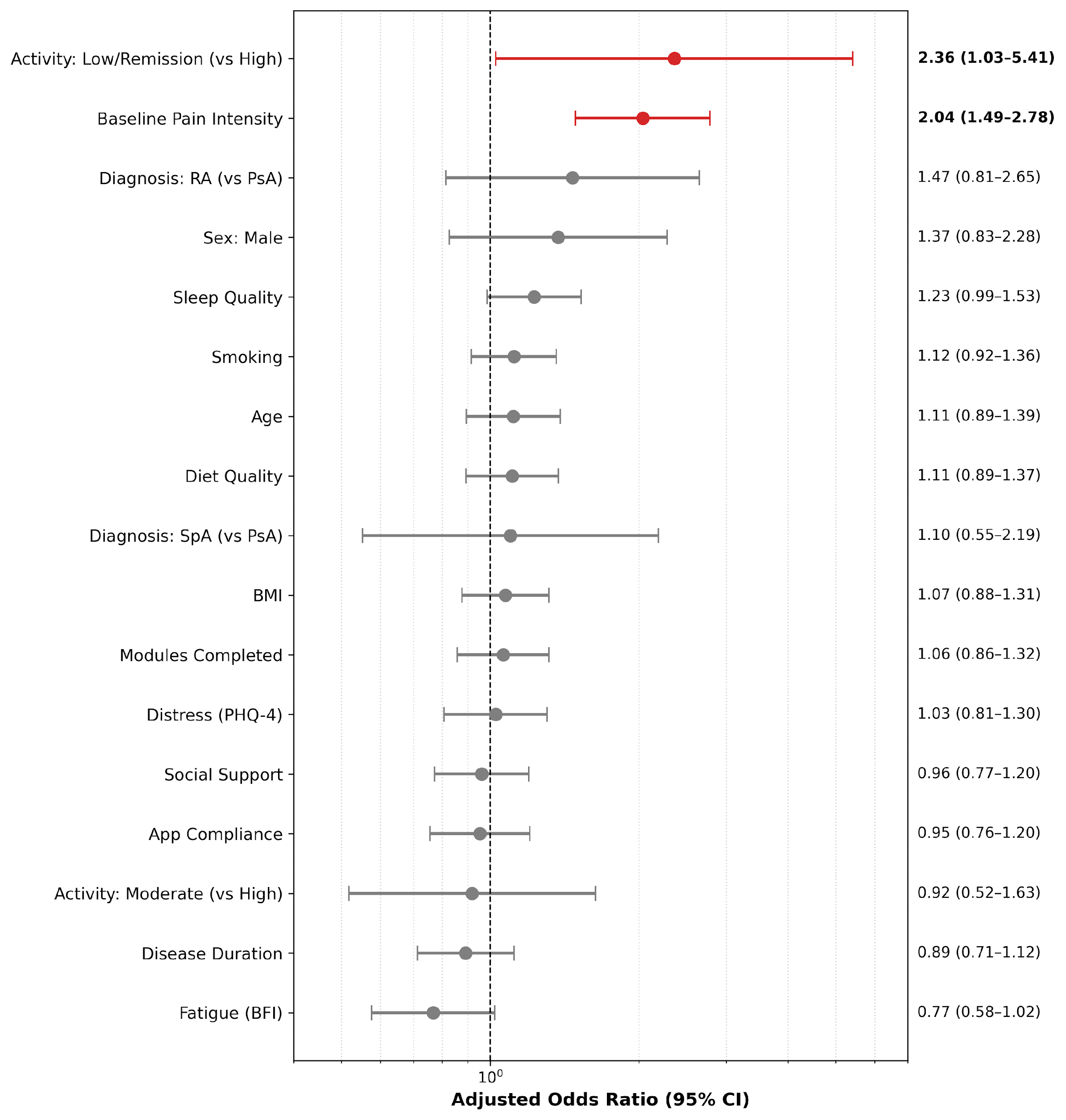
**Figure S**5


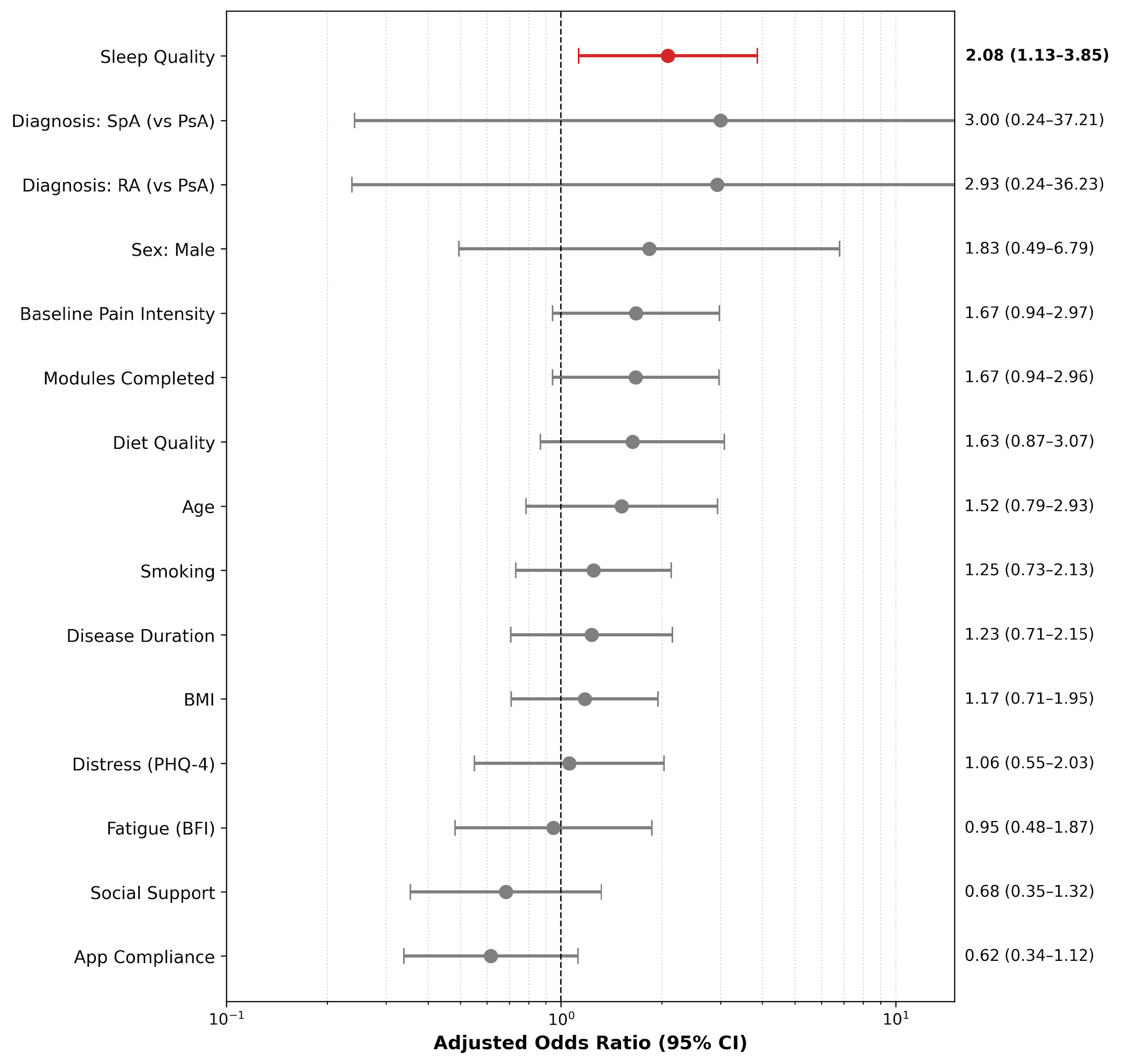
**Figure S**6
